# Lightweight frequency-aware hybrid vision transformer for fine-grained plant disease recognition

**DOI:** 10.3389/fpls.2026.1956762

**Published:** 2026-10-13

**Authors:** Kun Li, Li Dong, Jing Qian, Shuai Wang, Luo-Luo Jiang, Wen-Jing Li

**Affiliations:** 1 School of Life Science, Guizhou Normal University, Guiyang, China; 2 School of Information Management and Artificial Intelligence, Zhejiang University of Finance and Economics, Hangzhou, China; 3 School of Computer Science and Technology, Zhejiang University of Water Resources and Electric Power, Hangzhou, China

**Keywords:** dual-branch architecture, edge computing, lightweight model, plant disease recognition, ViTs

## Abstract

Plant diseases can substantially reduce crop yield and quality, while accurate disease recognition on edge devices remains challenging because images often contain subtle lesion textures and complex backgrounds. This study proposes a lightweight frequency-aware Dual-Embedding Hybrid Vision Transformer, termed DE-HVT, for fine-grained plant disease recognition. Here, “frequency-aware” does not denote an explicit or orthogonal separation into independent frequency bands. Instead, it refers to complementary frequency-biased processing by two spatially aligned embedding branches: OBPE preserves slowly varying structural cues, whereas the CDC-based TexPE branch has a zero response at zero spatial frequency under the linearised θ=1 analysis and therefore exhibits difference-sensitive, high-pass-like responses to lesion boundaries and textures. Their outputs are fused by element-wise addition before MHSA, while ConvFFN introduces local spatial filtering after global token interaction. Experiments were conducted on the Plant Pathology and PlantVillage datasets using the same training and evaluation protocols for all compared models. Across three independent runs, DE-HVT achieved a mean Top-1 accuracy of 93.51±0.44% and a mean macro-averaged F1 score of 93.64±0.40% on the more challenging Plant Pathology dataset. On the controlled-background PlantVillage dataset, all evaluated models achieved near-ceiling recognition performance. The model contains 4.10 million parameters and requires 0.85 GFLOPs. On a Snapdragon 865-based mobile platform, it used 46.86 MB of memory and recorded a maximum observed inference latency of 111.96 ms per image. These results indicate that DE-HVT provides a favourable balance between recognition performance and computational efficiency within the evaluated datasets and under the tested mobile-device conditions.

## 1 Introduction

Plant diseases represent a major constraint on agricultural production because they can reduce both crop yield and product quality. The increasing frequency of pathogen outbreaks, partly driven by climate change and intensive agricultural practices, has further strengthened the need for timely and reliable disease monitoring (Chakraborty, 2013; Singh et al., 2023). Conventional disease diagnosis generally relies on visual inspection by experienced agronomists or on laboratory analysis. Although these approaches can provide reliable results, they are often time-consuming, labour-intensive and difficult to implement consistently across large cultivation areas (Jogekar and Tiwari, 2021; Dang et al., 2024). The limited availability of qualified specialists also restricts the feasibility of continuous disease surveillance in orchards and other agricultural environments. Consequently, image-based plant disease recognition supported by lightweight computational models has attracted increasing attention for deployment on mobile devices, portable agricultural equipment and other edge platforms.

Early automated recognition methods were primarily based on hand-crafted colour, shape and texture descriptors. For example, support vector machines combined with lesion segmentation and manually designed colour and texture features have been used to identify grape leaf diseases (Padol and Yadav, 2016). Haralick texture descriptors have also been combined with conventional classifiers for leaf disease recognition (Das et al., 2020). These studies demonstrated that local visual characteristics can provide useful information for discriminating between plant diseases. However, the performance of such methods depends heavily on feature design and prior domain knowledge. Hand-crafted descriptors are also sensitive to variations in illumination, viewing conditions and background complexity, limiting their generalisation to uncontrolled agricultural environments.

Deep convolutional neural networks (CNNs) have become an important approach to image-based plant disease recognition because their local receptive fields and weight-sharing mechanisms are well suited to extracting colour, shape and texture features from plant leaves (He et al., 2016; Tan and Le, 2019). CNN-based models have been applied to disease classification, segmentation and detection in both controlled and natural environments (Dang et al., 2020; Li et al., 2020; Dang et al., 2024). Several lightweight or enhanced CNN architectures have also been developed for practical applications. MobileNet-based transfer-learning models, for instance, have been used to improve the identification of disease-relevant regions without substantially increasing computational cost (Chen et al., 2020). Other approaches have integrated multiple convolutional backbones and attention modules to improve disease recognition in grape leaves (Xie et al., 2020), while EfficientNet has been employed to achieve a more favourable balance between model capacity and computational complexity (Atila et al., 2021). Wavelet-based feature fusion and semi-supervised learning have also been investigated to improve recognition under conditions involving multi-scale disease patterns, limited training data and background interference (Panchananam et al., 2025; Zhao et al., 2025).

Although conventional CNNs are effective at extracting local features, they remain constrained by their local receptive fields. Capturing long-range dependencies often requires deeper architectures or additional operations, thereby increasing parameter redundancy and computational cost. This limitation is particularly significant when disease-related cues are distributed across different regions of a leaf or when the background contains visually similar structures. Vision Transformers (ViTs) offer a means of addressing this limitation by modelling relationships between image patches through self-attention (Dosovitskiy et al., 2021). Hierarchical and data-efficient Transformer architectures, including Swin Transformer and DeiT, have subsequently improved the applicability of Transformer-based models to visual recognition tasks (Liu et al., 2021; Touvron et al., 2021). In plant disease recognition, Transformer-based methods have been employed to model multi-scale lesions, suppress irrelevant background responses and enhance the representation of disease regions (Chang et al., 2024; Kunduracioglu and Pacal, 2024; Wang et al., 2024).

Fine-grained visual classification generally concerns the recognition of subordinate categories within a shared basic-level category and is characterised by small inter-class differences and substantial intra-class variation (Wei et al., 2022). In plant disease recognition, this setting arises when diseases affecting the same crop share similar overall leaf morphology but differ mainly in localised symptom regions. Differences in lesion shape, boundary definition, colour distribution and surface texture may be subtle across disease categories, whereas symptom appearance may vary considerably within the same category because of disease severity, illumination, leaf orientation and acquisition conditions. Distinguishing these categories therefore requires discriminative local symptoms to be represented together with their broader spatial context. The present study addresses this fine-grained recognition problem under the additional constraint of lightweight edge deployment. However, directly applying ViTs to plant disease recognition presents two related challenges. First, self-attention is computationally expensive because its complexity increases quadratically with the number of image tokens, limiting the deployment of standard ViTs on mobile and embedded devices. Secondly, patch partitioning and global feature aggregation may attenuate the representation of local edges, textures and other fine-grained patterns. This issue is particularly important for early-stage disease symptoms, for which distinctions between visually similar categories may depend on small lesion boundaries, spots or surface textures rather than on the overall structure of the leaf. Although CNN–Transformer hybrid models combine local and global representations, they differ in how these representations are constructed and integrated. Related plant disease studies have explored lightweight asymmetric hybrid architectures (Tian et al., 2026) and multimodal Transformer-based fusion for disease segmentation (Song et al., 2025). Among closely related lightweight hybrid models, MobileViT (Mehta and Rastegari, 2022) combines local convolutions with Transformer-based global processing and subsequently fuses the globally processed features with the block input. EdgeViT (Pan et al., 2022) organises local aggregation, sparse global attention and local propagation within a local–global–local bottleneck. DHVT (Lu et al., 2025) incorporates convolution into embedding and feed-forward processing, together with dynamic feature aggregation and head-token interaction.

DE-HVT differs in the specific construction and staging of its complementary representations. OBPE and CDC-based TexPE process corresponding overlapping image regions in parallel: OBPE provides overlapping structural embeddings, while TexPE encodes local intensity differences and texture variations within each patch. Their spatially aligned outputs are fused by element-wise addition before the first MHSA operation, without increasing the token count or embedding dimension at fusion. ConvFFN subsequently introduces local interactions between neighbouring tokens after MHSA in each encoder block. This arrangement combines within-patch texture-sensitive token construction before global interaction with token-grid spatial refinement afterwards, defining the specific architectural distinction of DE-HVT within the broader family of lightweight hybrid models.

The importance of preserving local details can also be understood from a frequency-domain perspective. Low-frequency components generally represent smooth structural information, such as global contours and broad colour distributions, whereas mid- and high-frequency components are more closely associated with edges, boundaries and fine textures. As illustrated in **Figure 1**, the evaluated apple disease categories share similar overall leaf morphology, whereas their differences are concentrated mainly in localised symptom regions. These symptoms may differ in lesion shape, boundary sharpness, colour distribution and surface texture. Such small inter-class differences provide the principal discriminative cues for fine-grained disease recognition. Therefore, preserving lesion boundaries, colour transitions and texture variations is important for distinguishing visually similar disease categories. Classical texture operators and local binary patterns have demonstrated the discriminative importance of local spatial variations in image analysis (Manjunath and Ma, 1996; Ojala et al., 2002). CNNs can capture such variations through convolutional operations, although fine details may become less prominent as features are progressively transformed through deeper layers. Similarly, global self-attention aggregates information across spatial positions and may reduce the relative prominence of local high-frequency details. Central difference convolution provides a computationally efficient means of enhancing local contrast and structural variation by modelling differences between a central pixel and its neighbouring pixels (Su et al., 2021). It is therefore well suited to extracting lesion-related edges and textures during the initial feature-embedding stage.

**Figure 1:**
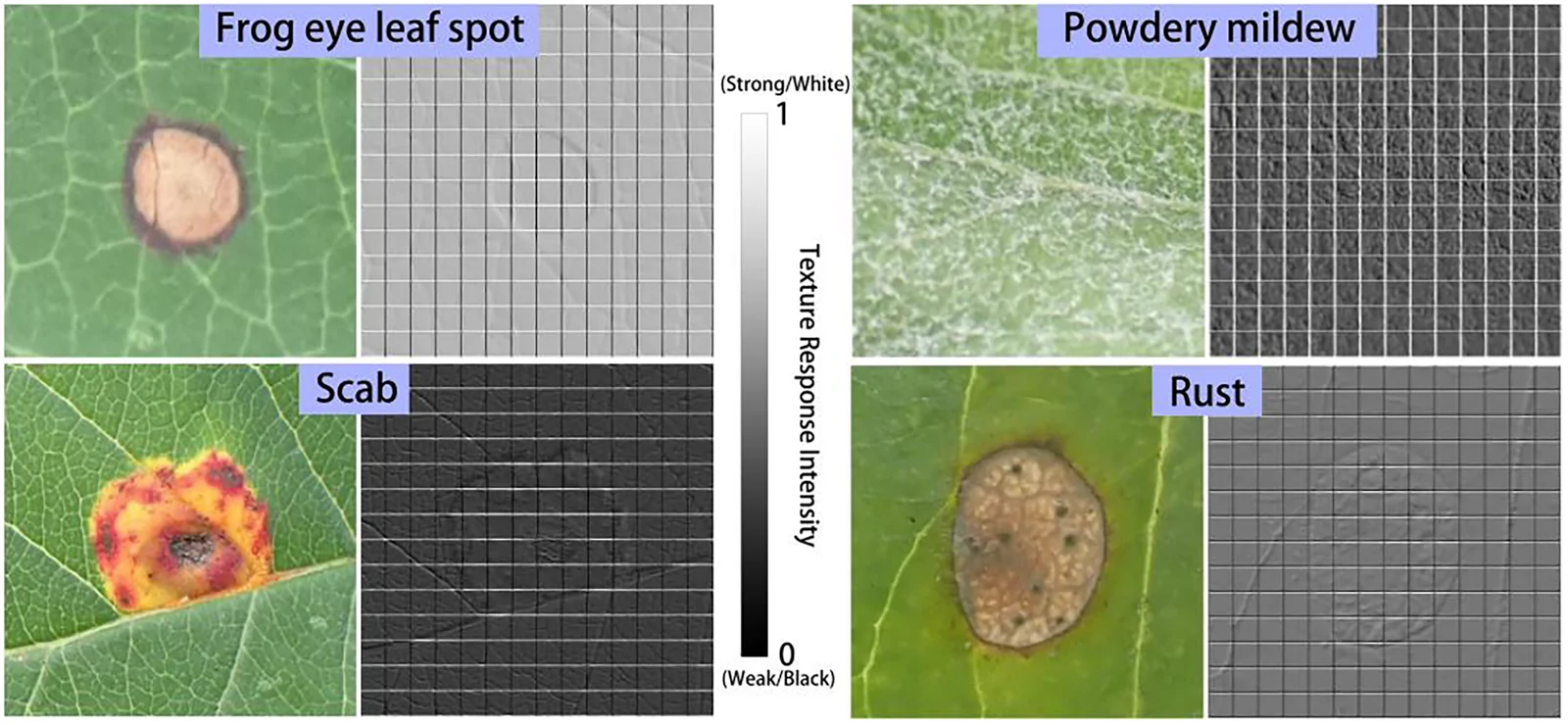
Examples of lesion shapes and texture patterns associated with four common apple leaf diseases. The colour intensity represents the magnitude of the texture response.

To address this gap, this study proposes DE-HVT, a lightweight frequency-aware hybrid Vision Transformer organised as a staged local–global–local processing pipeline. At the tokenisation stage, OBPE and TexPE process the same input in parallel using the same window arrangement and sliding stride, thereby producing spatially aligned token sequences. OBPE uses overlapping convolutional projection to preserve continuity between neighbouring image regions and broader structural cues. In contrast, TexPE applies CDC within the corresponding image regions to emphasise local intensity differences, lesion boundaries and texture variations, followed by channel compression, normalisation and linear projection. Both branches project their outputs to the same embedding dimension, and the resulting token sequences are fused by element-wise addition before MHSA.

MHSA subsequently models long-range relationships among the fused image tokens. After global interaction, ConvFFN rearranges the token sequence into a two-dimensional feature map and applies pointwise, depthwise and pointwise convolutions to introduce local spatial interactions within the Transformer block. The architectural contribution of DE-HVT therefore lies not in claiming CDC, overlapping embedding, self-attention or depthwise convolution as individually new operations, but in assigning them complementary roles and placing them at different stages of a compact processing sequence. This design introduces texture sensitivity before global interaction and local spatial filtering after it, without requiring a separate heavy CNN backbone, while retaining a fixed token-embedding dimension during dual-branch fusion.

Based on this design, the principal contributions of this study are threefold. First, a dual-embedding tokenisation strategy is developed to construct spatially aligned structural and texture-sensitive representations before global self-attention. Second, a staged local–global–local processing scheme is established by combining dual-branch embedding, MHSA and ConvFFN within a compact six-block Transformer, thereby introducing local inductive biases both before and after global token interaction. Third, extensive component, window-overlap and network-depth ablations are conducted to examine the individual and combined contributions of the principal architectural components, while comparisons on Plant Pathology and PlantVillage evaluate recognition performance, computational complexity and mobile-device feasibility.

In this study, frequency-aware complementary processing refers to learned module-level biases toward spatial variations at different scales; it does not denote an explicit, orthogonal or lossless decomposition into independent frequency bands. Slowly varying spatial components are associated primarily with broad leaf structure and colour distribution, whereas more rapidly varying components are associated with lesion boundaries, small spots and local textures. TexPE introduces a theoretically interpretable difference-sensitive bias through CDC, while OBPE retains broader structural information. Because the two branch outputs are subsequently fused, DE-HVT should not be interpreted as maintaining strictly separated frequency bands.

## 2 Materials and methods

### 2.1 Datasets

Two publicly available plant disease image datasets were used to evaluate DE-HVT under complementary recognition conditions. Plant Pathology provided the primary single-crop fine-grained evaluation under natural orchard conditions, whereas PlantVillage provided a broader multi-crop evaluation under relatively controlled conditions. Representative images from the two datasets are shown in **Figure 2**.

**Figure 2:**
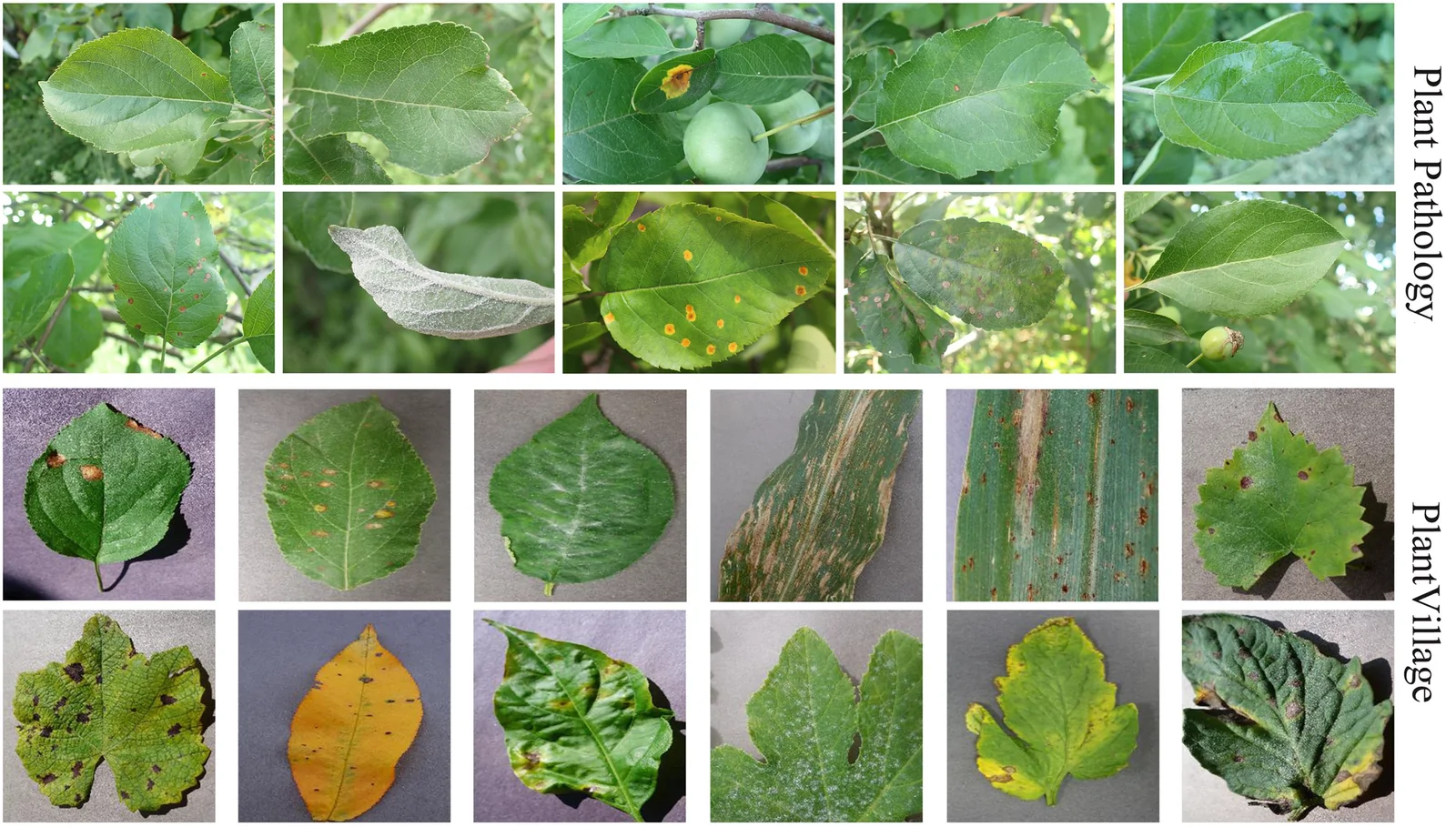
Representative samples from the Plant Pathology and PlantVillage datasets. The Plant Pathology samples contain natural orchard backgrounds and variable imaging conditions, whereas the PlantVillage samples were acquired primarily under relatively controlled conditions.

#### 2.1.1 Plant Pathology dataset

The Plant Pathology dataset used in this study was derived from the expanded Plant Pathology 2021–FGVC8 challenge dataset, which was developed from the orchard image collection originally described by Thapa et al. (2020). The images were collected at Cornell AgriTech in Geneva, New York, using handheld cameras under natural orchard conditions. Consequently, the dataset contains substantial variation in illumination, leaf orientation, background composition and disease severity.

The initial subset used in this study comprised 18,632 images from six categories: healthy, scab, rust, powdery mildew, frogeye leaf spot and complex disease. The complex-disease category contained images showing heterogeneous combinations of symptoms associated with more than one disease. Consequently, it did not represent a single disease entity with a consistent visual definition, and its symptoms could partially overlap with those of several individual disease categories. Treating these heterogeneous combinations as one mutually exclusive class would therefore be inconsistent with the single-label classification formulation adopted in this study and with the objective of distinguishing well-defined fine-grained disease categories. For this reason, the 1602 complex-disease images were excluded, leaving 17030 images from five clearly defined categories. Accordingly, the reported Plant Pathology results refer specifically to this five-class single-label setting and do not evaluate the recognition of leaves affected by multiple concurrent diseases.

Plant Pathology provides a particularly relevant fine-grained recognition setting because the evaluated categories consist of healthy apple leaves and four disease conditions sharing the same host species and similar overall leaf morphology. The disease categories may exhibit partially overlapping local symptoms, including small spot-like or irregular lesions, similar colour ranges, and variations in lesion-boundary sharpness and surface texture. Consequently, discrimination between the disease categories depends less on global leaf structure and more on subtle differences in lesion shape, boundary characteristics, colour distribution and texture. At the same time, symptom appearance may vary within the same category because of disease severity, illumination, leaf orientation and background composition. The dataset therefore exhibits both small inter-class differences and substantial intra-class variation, which are characteristic of fine-grained visual classification. Early-stage lesions may also occupy only a small proportion of the leaf surface, requiring the model to preserve local symptom details while using the broader leaf context. After all images were resized to 224×224pixels, each disease category was independently divided into training, validation and test subsets using a 70:15:15 ratio. The category-specific subsets were then combined to form the final dataset partitions. The resulting aggregate partition is summarised in **Table 1**, and all reported results were calculated using the test subset.

**Table 1:** Dataset composition and image-level partitioning.

Dataset	Total images	Classes	Plant species	Native resolution	Input resolution	Train	Validation	Test
Plant Pathology	17,030	5	Apple	4000 × 2672	224 × 224	11,913	2,556	2,561
PlantVillage	54,305	38	14 plant species	256 × 256	224 × 224	38,014	8,146	8,145

#### 2.1.2 PlantVillage dataset

The PlantVillage dataset was originally introduced as an open repository of plant-health images to support the development of image-based plant disease diagnosis systems (Hughes and Salathé, 2015; Mohanty et al., 2016). It contains 54,305 images from 38 crop–disease categories, covering healthy and diseased leaves from 14 plant species. According to the official dataset description, detached leaves were photographed against grey or black background sheets under relatively controlled acquisition conditions. Several images were typically acquired from the same physical leaf while the leaf was rotated, and different regions were photographed when a leaf was too large to be captured in a single image. The images were subsequently cropped and oriented consistently. For the diseased categories, the selected leaves generally exhibited visually apparent symptoms and were curated according to expert diagnoses. These acquisition and processing procedures provide clearly visible leaf regions, readily observable disease characteristics and limited background interference, but they may also introduce visual similarity among images and dataset-specific background or composition cues. Compared with the Plant Pathology dataset, these standardised imaging conditions provide more distinct crop–disease visual cues. The 38-class PlantVillage task is not uniformly fine-grained because differences between crop species can provide additional global recognition cues. Nevertheless, crops such as apple, corn, grape, potato and tomato contain multiple disease categories sharing the same general leaf structure. Discrimination within these crops still depends on local differences in lesion shape, boundary definition, colour and texture. PlantVillage therefore provides complementary within-crop fine-grained comparisons, although its overall multi-crop setting is less challenging in this respect than the single-crop Plant Pathology task.

The PlantVillage dataset was used to evaluate whether DE-HVT could maintain reliable recognition performance across multiple plant species and disease categories under relatively controlled imaging conditions. After all images were resized to 224×224 pixels, each crop–disease category was independently divided into training, validation and test subsets using a 70:15:15 ratio. The category-specific subsets were then combined to form the final dataset partitions. The resulting aggregate partition is summarised in **Table 1**. A fixed random seed was used to generate the partition, and the same partition was adopted for all compared models. Given the image-level partitioning protocol, visually similar but non-identical images may occur in different subsets. All reported performance values were calculated using the test subset.

### 2.2 DE-HVT architecture

DE-HVT is a lightweight hybrid Vision Transformer designed to preserve lesion-related local details while retaining the global contextual modelling capability provided by self-attention. The overall architecture is illustrated in **Figure 3** and comprises four principal components: overlapping-window patch embedding (OBPE), texture-aware patch embedding (TexPE), multi-head self-attention (MHSA) and a convolutional feed-forward network (ConvFFN). OBPE and TexPE operate in parallel to construct complementary initial feature representations. OBPE primarily captures broad structural and semantic information, whereas TexPE emphasises local contrast, lesion boundaries and texture variations. The features extracted by the two branches are projected into the same embedding dimension and fused through element-wise addition before global feature modelling. The resulting representation is subsequently processed by a stack of Transformer encoder blocks comprising MHSA and ConvFFN, followed by global average pooling and a linear classification head. The frequency-aware property of DE-HVT arises from the complementary processing behaviours of these modules and does not imply an explicit or orthogonal decomposition of the input into predefined frequency bands.

**Figure 3:**
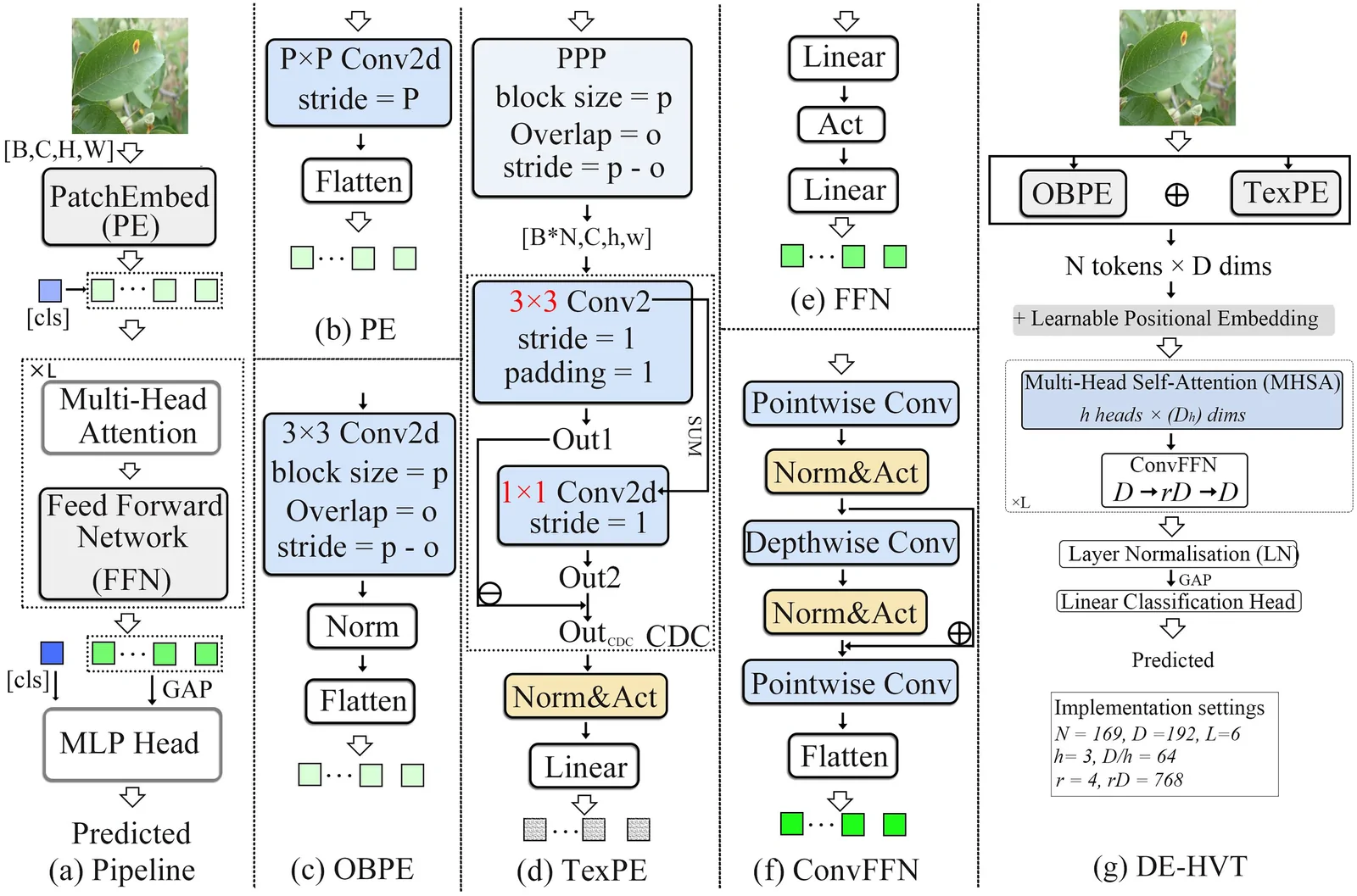
Overall architecture of DE-HVT and comparison with a generic Vision Transformer. **(a)** Generic Vision Transformer pipeline. **(b)** Conventional non-overlapping patch embedding. **(c)** Overlapping Block Patch Embedding (OBPE). **(d)** Texture-aware Patch Embedding (TexPE). **(e)** Conventional feed-forward network. **(f)** Convolutional Feed-Forward Network (ConvFFN). **(g)** Overall DE-HVT architecture, in which the OBPE and TexPE outputs are fused by element-wise addition and processed by repeated Transformer encoder blocks, global average pooling and a linear classification head. The symbols ⊕ and ×L denote element-wise addition and repetition of the encoder block, respectively. In panel **(g)**, N, D, L, h, and r denote the number of tokens, embedding dimension, number of encoder blocks, number of attention heads, and ConvFFN expansion ratio, respectively; D/h is the per-head dimension, and the adopted implementation settings are listed in the inset.

The final DE-HVT configuration follows a single-resolution processing pipeline comprising three functional stages. First, a 224×224 RGB image is processed in parallel by OBPE and TexPE using spatially aligned 17×17 embedding windows with a one-pixel overlap and a stride of 16. Both branches generate 169 tokens with an embedding dimension of 192, and their outputs are fused by element-wise addition. Second, the fused token sequence is processed by six Transformer encoder blocks, each containing three-head MHSA and ConvFFN. Finally, layer normalisation, global average pooling and a linear classifier are applied to obtain the class prediction. The spatial token resolution and embedding dimension remain unchanged throughout the six encoder blocks. To further clarify the interaction among the principal components, the end-to-end forward propagation of DE-HVT is summarised in **Algorithm 1**. Here, P denotes the learnable positional embedding, K=5 for Plant Pathology and K=38 for PlantVillage.

Algorithm 1

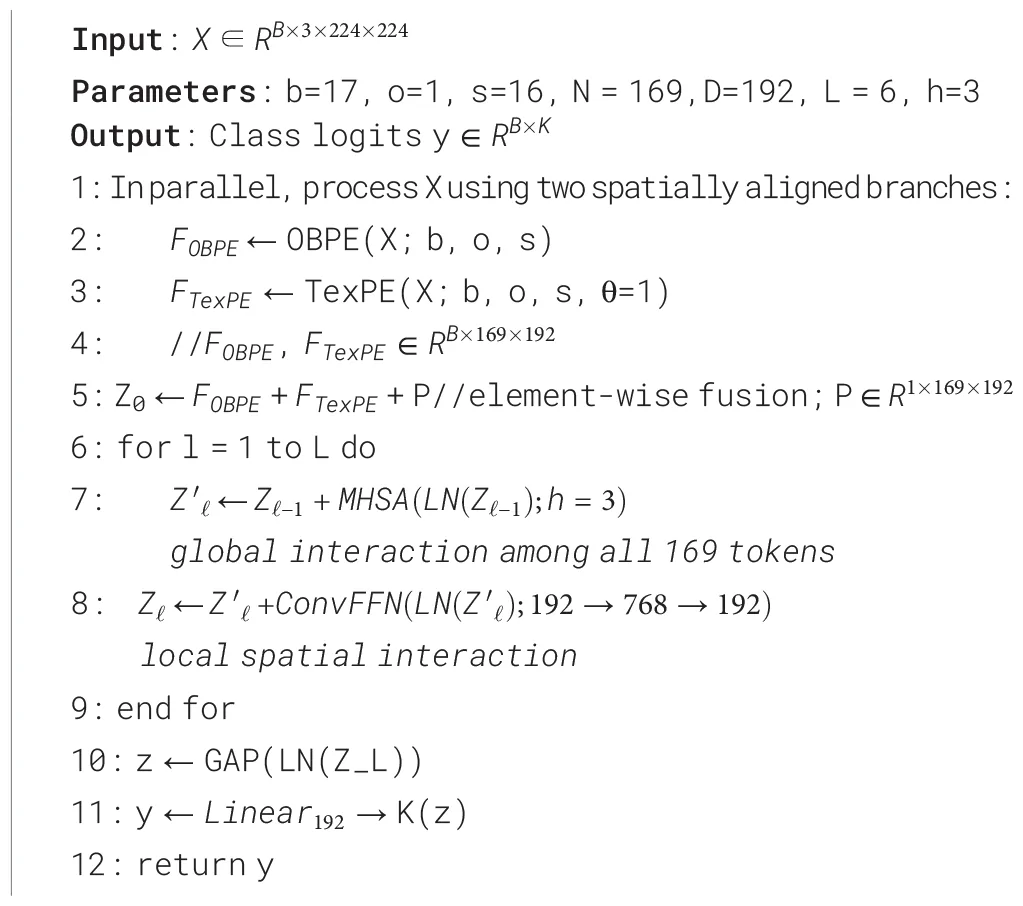

### 2.3 Dual-branch patch embedding

The dual-branch patch-embedding module is designed to construct complementary low-level representations from the input image. As shown in **Figure 4**, the input image is processed simultaneously by the Overlapping Block Patch Embedding (OBPE) branch and the Texture-aware Patch Embedding (TexPE) branch. The two branches use the same patch size and sliding stride so that their outputs remain spatially aligned. The corresponding operations are illustrated in **Figures 3c, d**, respectively. OBPE mainly captures the structural and semantic information associated with the overall leaf appearance, whereas TexPE enhances the representation of local contrast, lesion boundaries and fine-grained texture variations. After projection to the same embedding dimension, the outputs of the two branches are combined by element-wise addition.

**Figure 4:**
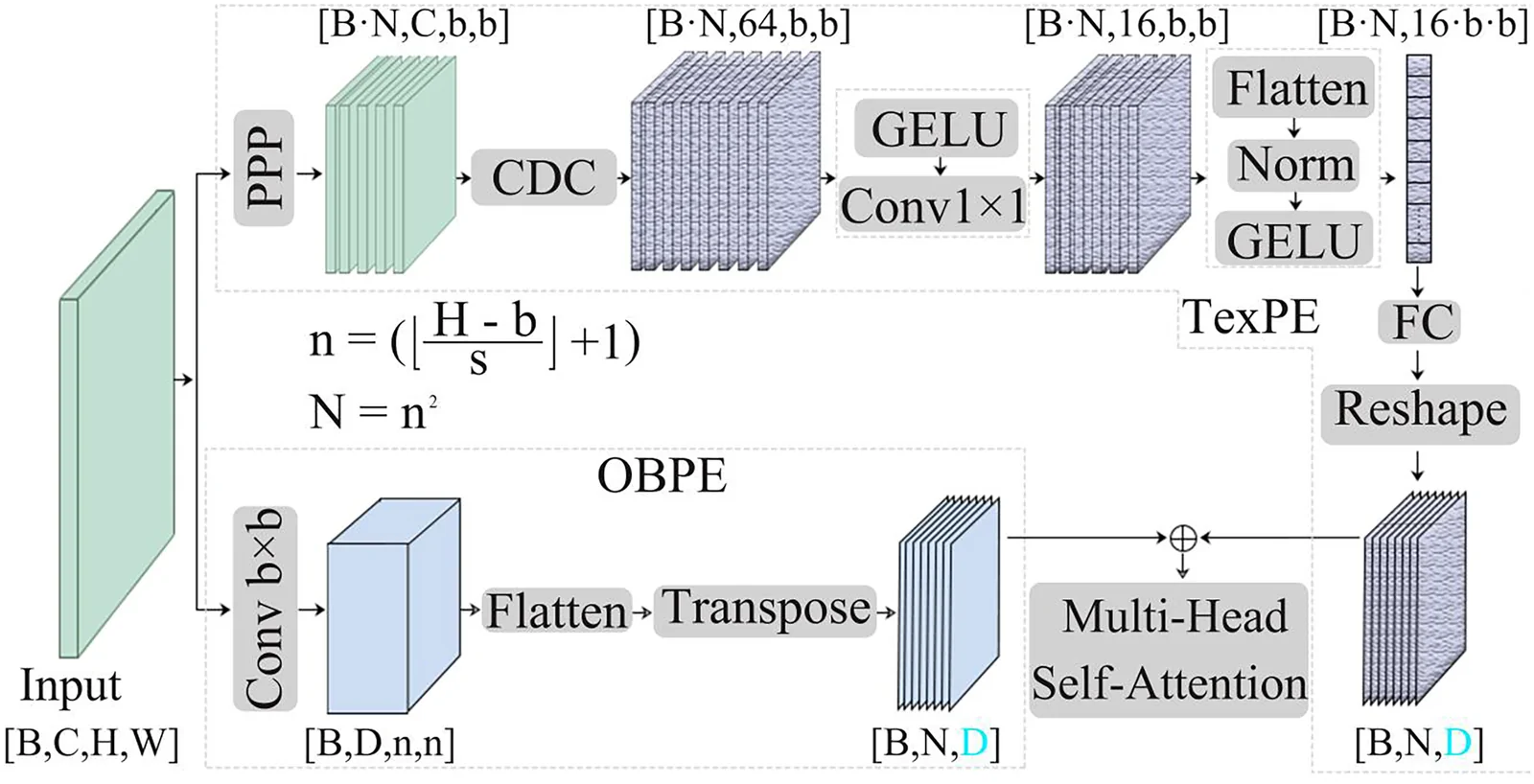
Detailed architecture of the dual-branch patch-embedding module in DE-HVT. The input image is processed in parallel by the Texture-aware Patch Embedding (TexPE) branch (upper path) and the Overlapping Block Patch Embedding (OBPE) branch (lower path). The TexPE branch consists of physical patch partitioning, Central Difference Convolution (CDC), 1×1 convolution, feature normalisation and linear projection, whereas the OBPE branch performs overlapping convolutional projection followed by flattening and transposition. The outputs of the two branches are aligned in both spatial ordering and embedding dimension and are combined by element-wise addition. A learnable positional embedding, omitted from the diagram for clarity, is subsequently added before the fused sequence is passed to MHSA. Here, ⊕ denotes element-wise addition. In the final configuration, b=17, o=1, s=16, N=169 and D=192.

#### 2.3.1 Overlapping block patch embedding

As illustrated in **Figure 3c**, OBPE replaces the conventional non-overlapping patch partitioning used in the standard Vision Transformer. Given an input image $\text{X}\in {\mathbb{R}}^{\text{B}\times \text{C}\times \text{H}\times \text{W}}$, where B, C, H and Wdenote the batch size, number of input channels, image height and image width, respectively, a convolutional window of size b is applied with an overlapping sliding strategy. If o denotes the overlap between two neighbouring windows, the sliding stride s is defined as:

(1) s=b−o

The convolutional projection and feature normalisation can then be expressed as:

(2) $${\text{V}}_{\text{OBPE}}=\text{BN}[\text{Conv}2\text{d}(\text{X};{\text{W}}_{\text{OBPE}},\text{kernel}=\text{b},\text{stride}=\text{s},\text{ padding}=0)]$$

where ${\text{W}}_{\text{OBPE}}$ represents the learnable convolutional weights and BN(·) denotes batch normalisation. The resulting feature map is flattened along its spatial dimensions and transposed to form a sequence of patch embeddings:

(3) $${\text{F}}_{\text{OBPE}}=\text{Transpose}[\text{Flatten}({\text{V}}_{\text{OBPE}},2),1,2]$$

In the final implementation, the input has C=3 channels, and OBPE uses a 17×17 convolution with a stride of 16, zero padding and 192 output channels. For a 224×224 input, the resulting feature map has a spatial size of 13×13. After flattening and transposition, the OBPE output is $F_{\text{OBPE}}\in {\mathbb{R}}^{B\times 169\times 192}$.

Compared with conventional non-overlapping patch embedding, the overlapping operation allows neighbouring image regions to share local information. This helps reduce discontinuities at patch boundaries and provides a more continuous representation of leaf contours, colour transitions and larger-scale lesion structures. The subsequent batch normalisation further stabilises the projected features before they are passed to the fusion stage.

#### 2.3.2 Texture-aware patch embedding

Although OBPE provides an effective representation of the overall leaf structure, convolutional projection may weaken subtle local variations during the initial embedding process. This is particularly relevant to plant disease recognition, because early-stage symptoms may be represented by small lesions, weak intensity changes or irregular texture patterns. Therefore, TexPE introduces Central Difference Convolution (CDC) to enhance the extraction of local structural information (Yu et al., 2020), as shown in **Figure 3d**.

To ensure spatial correspondence with the OBPE branch, the input image is first divided into overlapping physical patches using the same window size b and sliding stride s:

(4) $${\text{U}}_{\text{patch}}={\text{Unfold}}_{\text{H}}({\text{Unfold}}_{\text{W}}(\text{X},\text{b},\text{s}),\text{b},\text{s})$$

The unfolded patches are permuted and reshaped into a batch of local patch regions:

(5) $${\text{U}}_{\text{PPP}}=\text{Reshape}[\text{Permute}({\text{U}}_{\text{patch}})]$$

where ${\text{ U}}_{\text{PPP}}\;$denotes the output of the Physical Patch Partition operation. **Equations 1**–**5** define the overlapping stride, OBPE projection and tokenisation, and TexPE physical-patch partitioning and reshaping operations. For a 3×3 convolutional kernel, the conventional convolutional response is first calculated as:

(6) $${\text{C}}_{\text{normal}}=\text{ Conv}2\text{d}({\text{U}}_{\text{PPP}};{\text{W}}_{\text{CDC}};\text{ stride }=\;1;\text{ padding }=\;1)$$

The sum of the kernel weights over the spatial dimensions is defined as:

(7) $${\text{W}}_{\text{sum}}\text{ = }\sum_{\text{p=}0}^{2}\sum_{\text{q=}0}^{2}{\text{W}}_{\text{CDC[:,:,p,q].}}$$

The CDC response is then obtained by subtracting the response generated by the summed kernel from the conventional convolutional response:

(8) $${\text{C}}_{\text{CDC}}={\text{C}}_{\text{normal}}-\text{θ Conv}2\text{d}({\text{U}}_{\text{PPP}};{\text{W}}_{\text{sum}},\text{kernel}=1,\text{stride}=1,\text{padding}=0)$$

The frequency-aware interpretation of CDC can be explained through its spatial-frequency response. For clarity, we consider a single input–output channel pair and omit the channel indices, bias, normalisation and nonlinear activation. Boundary effects introduced by finite-image padding are also excluded from this linearised analysis. Let w(δ) denote the convolutional coefficient at spatial offset δ∈K, where Kdenotes the kernel support. The conventional convolutional response is defined in Equation 6 while the summed kernel weights used in CDC are defined in Equation 7. The corresponding spatial-frequency response of the conventional convolution is:

(9) $${\text{H}}_{\text{normal}}(\text{ω})\;=\;\sum_{\text{δ}\in \text{K}}\text{w}(\text{δ}){\text{e}}^{-{\text{jω}}^{\text{T}}\text{δ}}$$

According to the CDC formulation in Equation 8, its response for a general central-difference coefficient θ is:

(10) $${\text{H}}_{\text{CDC}}(\text{ω})\;={\text{ H}}_{\text{normal}}(\text{ω})-{\text{θH}}_{\text{normal}}(0)=\sum_{\text{δ}\in \text{K}}\text{w}(\text{δ})({\text{e}}^{-{\text{jω}}^{\text{T}}\text{δ}}-\text{θ})$$

At zero spatial frequency:

(11) $${\text{H}}_{\text{CDC}}(0)\;=\;(1-\text{θ}){\text{H}}_{\text{normal}}(0)$$

The TexPE implementation used in this study adopts θ=1; therefore, $H_{\text{CDC}}(0)=0$. Under the assumptions of the linearised analysis, CDC suppresses the response to spatially constant components. For small non-zero spatial frequencies:

(12) $${\text{e}}^{-{\text{jω}}^{\text{T}}\text{δ}}-1\;\approx \;-{\text{jω}}^{\text{T}}\text{δ}$$

and consequently:

(13) $${\text{H}}_{\text{CDC}}(\text{ω})\approx -{\text{jω}}^{\text{T}}\sum_{\text{δ}\in \text{K}}\text{w}(\text{δ})\text{δ}+\text{O}(\|\text{ω}{\|}^{2})$$

**Equations 9**–**13** describe the spatial-frequency responses of the conventional convolution and CDC, together with the corresponding zero- and small-frequency approximations. This approximation shows that the CDC response depends on local spatial variation rather than on absolute intensity alone, providing a difference-sensitive, high-pass-like tendency that is suitable for representing lesion boundaries, small spots and texture transitions. However, this theoretical property does not imply that CDC is an ideal high-pass filter or that TexPE explicitly separates the input into independent frequency bands. The convolutional weights are learned from data, and the complete TexPE branch also contains finite-support convolution, padding, nonlinear activation, channel compression, normalisation and linear projection. The analysis therefore explains the enhanced sensitivity of TexPE to fine spatial variations without claiming an exact or orthogonal frequency decomposition.

The CDC features are subsequently activated using the Gaussian Error Linear Unit (GELU):

(14) $${\text{F}}_{\text{CDC}}=\text{ GELU}({\text{C}}_{\text{CDC}})$$

A 1×1 convolution is then used to compress the channel dimension:

(15) $${\text{F}}_{\text{comp}}=\text{ Conv}2{\text{d}}_{1\times 1}({\text{F}}_{\text{CDC}};{\text{W}}_{\text{comp}})$$

Finally, the compressed features are flattened, normalised and linearly projected into the required embedding dimension:

(16) $${\text{F}}_{\text{TexPE}}={\text{W}}_{\text{fc}}\text{GELU}[\text{LN}(\text{Flatten}({\text{F}}_{\text{comp}}))]+{\text{b}}_{\text{fc}}$$

Here, LN(·) denotes layer normalisation, while $W_{\text{fc}}$ and $b_{\text{fc}}$ are the weights and bias of the linear projection layer, respectively.

**Equations 14**–**16** define the activation, channel compression, and final projection used to obtain the TexPE representation. In the final TexPE configuration, each physical patch has a spatial size of 17×17. CDC uses a 3×3 kernel with stride 1 and padding 1 to project the three input channels to 64 channels. A subsequent 1×1 convolution compresses the representation from 64 to 16 channels. The resulting 16×17×17=4624 features are flattened and linearly projected to the 192-dimensional token space, producing ${\text{F}}_{\text{TexPE}}\in {\mathbb{R}}^{\text{B}\times 169\times 192}$.

#### 2.3.3 Feature fusion

The outputs of the OBPE and TexPE branches are projected to the same embedding dimension and retain the same spatial ordering. They are therefore fused directly by element-wise addition:

(17) $${\text{F}}_{0}={\text{F}}_{\text{OBPE}}+{\text{F}}_{\text{TexPE}}$$

Before entering the first Transformer encoder block, a learnable positional embedding $P\in {\mathbb{R}}^{1\times 169\times 192}$ is added to the fused representation. The position-augmented sequence is therefore denoted by $Z_{0}=F_{0}+P$, where $F_{0}$ is the fused dual-branch representation defined in Equation 17, and $Z_{0}$ is the actual input to the first Transformer encoder block. This fusion operation preserves the complementary information extracted by the two branches: OBPE contributes broader structural and semantic cues, whereas TexPE provides enhanced local contrast and texture information. The position-augmented representation is then forwarded to MHSA for global contextual modelling.

### 2.4 Self-attention-based global feature modelling

Following dual-branch patch embedding, the fused representation is processed by Multi-Head Self-Attention (MHSA) to capture global contextual relationships among the embedded image regions. The position of MHSA within DE-HVT is shown in **Figure 3g**, while its connection to the outputs of OBPE and TexPE is illustrated in **Figure 4**. The implementation follows the standard Transformer attention mechanism (Vaswani et al., 2017; Dosovitskiy et al., 2021).

Given an input sequence $\text{ X}\in {\mathbb{R}}^{\text{B}\times \text{N}\times \text{D}}$, layer normalisation is first applied, after which the features are jointly projected and reshaped into query, key and value tensors:

(18) $$\text{Q},\text{K},\text{V }=\text{ Reshape}[\text{LN}(\text{X}){\text{W}}_{\text{qkv}}+{\text{b}}_{\text{qkv}}]$$

for the i-th attention head, scaled dot-product attention is calculated as:

(19) $${\text{head}}_{\text{i}}=\text{ Softmax}(\frac{{\text{Q}}_{\text{i}}{\text{K}}_{\text{i}}^{T}}{\sqrt{{\text{d}}_{\text{h}}}}){\text{V}}_{\text{i}}$$

where $({\text{d}}_{\text{h}}=\text{D}/\text{h})\;$is the feature dimension of each attention head and h is the number of heads. The outputs of all heads are concatenated and linearly projected:

(20) $$\text{MHSA}(\text{X})\;=\text{ Concat}({\text{head}}_{1},\ldots ,{\text{head}}_{\text{h}}){\text{W}}_{\text{O}}+{\text{b}}_{\text{O}}$$

**Equations 18**–**20** define the query–key–value projection, per-head attention, and multi-head output projection, respectively. In the final configuration, D=192 and h=3, resulting in a per-head dimension of $d_{h}=64$. MHSA is applied globally across all 169 tokens; therefore, DE-HVT does not employ a separate local attention-window size.

A residual connection is applied to the MHSA output, which is subsequently passed to the ConvFFN sublayer. This operation enables information exchange among spatially separated image regions and provides global contextual features for subsequent local feature refinement.

### 2.5 Convolutional feed-forward network

The conventional feed-forward network processes each image embedding independently using two linear transformations, as illustrated in **Figure 3e**, and therefore does not explicitly model local spatial interactions. Previous studies have introduced convolutional operations into Transformer feed-forward networks to strengthen locality while retaining the global representation produced by self-attention (Li et al., 2023; Lu et al., 2025). Based on this principle, DE-HVT replaces the conventional feed-forward network with a Convolutional Feed-Forward Network (ConvFFN). Its position within the DE-HVT encoder is shown in **Figure 3g**, while its detailed structure is presented in **Figure 5**.

**Figure 5:**
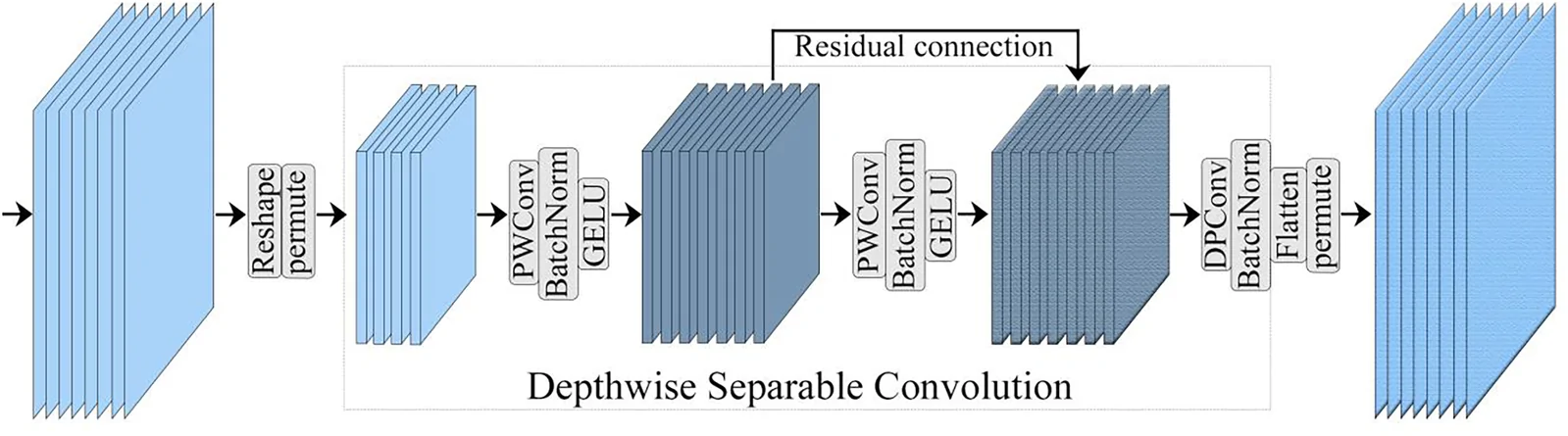
Detailed structure of the Convolutional Feed-Forward Network (ConvFFN). The input token sequence is rearranged into a two-dimensional feature map and processed sequentially by a  1×1 pointwise convolution, a  3×3 depthwise convolution with an internal residual connection, and a second  1×1 pointwise convolution. Batch normalisation and GELU activation are applied after the first pointwise convolution and the depthwise convolution, whereas only batch normalisation is applied after the final pointwise projection. The resulting feature map is flattened and rearranged back into a token sequence. This structure introduces local spatial interactions and channel-wise feature mixing while preserving the input–output embedding dimension. The final ConvFFN configuration adopts a 192→768→192 channel arrangement.

As illustrated in **Figure 3f** and **Figure 5**, the input sequence is first rearranged into a two-dimensional feature map according to its original spatial ordering. A 1×1 pointwise convolution expands the channel dimension and enables information interaction across channels. This operation is followed by batch normalisation and GELU activation:

(21) $${\text{D}}_{\text{c}1}=\text{GELU}[\text{BN}(\text{PWConv}(\text{X}))]$$

where  PWConv(·) denotes pointwise convolution and ${\text{ D}}_{\text{c}1}\;$is the expanded intermediate representation. A 3×3 depthwise convolution is then applied independently to each channel to capture local spatial relationships. An internal residual connection is introduced between the input and output of this operation:

(22) $${\text{D}}_{\text{c}2}=\text{ GELU}[\text{BN}(\text{DWConv}({\text{D}}_{\text{c}1}))]+{\text{D}}_{\text{c}1}$$

where  DWConv(·) denotes depthwise convolution. Finally, a second  1×1 pointwise convolution projects the features back to the original embedding dimension:

(23) $${\text{F}}_{\text{ConvFFN}}=\text{BN}[\text{PWConv}({\text{D}}_{\text{c}2})]$$

**Equations 21**–**23** define the channel expansion, depthwise spatial filtering with an internal residual connection, and final channel projection in ConvFFN. ConvFFN uses a channel-expansion ratio of four. Specifically, the first 1×1 pointwise convolution expands the channel dimension from 192 to 768. The subsequent 3×3 depthwise convolution uses stride 1, padding 1 and 768 groups, after which the final 1×1 pointwise convolution projects the representation from 768 back to 192 channels.

The resulting feature map is flattened and transposed back into a sequence before being combined with the encoder input through the outer residual connection. The pointwise convolutions provide channel-wise feature interaction, whereas the depthwise convolution introduces local spatial modelling with limited computational overhead. ConvFFN therefore complements the global contextual relationships learned by MHSA with local spatial information, helping to preserve lesion boundaries and texture variations during feature transformation.

### 2.6 Experimental design

The experiments were designed to evaluate DE-HVT from three complementary perspectives: recognition performance and computational efficiency, the contributions of the principal architectural components, and feasibility for deployment on a resource-constrained mobile device. All models were evaluated using the same dataset partitions and evaluation procedures to ensure a consistent comparison. Classification performance was assessed on both the Plant Pathology and PlantVillage datasets, whereas the ablation and frequency-domain analyses were conducted on the more challenging Plant Pathology dataset.

#### 2.6.1 Implementation and training configuration

Model training was conducted on a server equipped with a single NVIDIA A800 GPU with 80 GB of memory and running Ubuntu 22.04. The models were implemented in Python 3.10 using PyTorch. Server-side inference latency was measured on the same A800 GPU using PyTorch with a batch size of 256, and the reported values represent the average inference latency per image under batched GPU execution. All models were trained from scratch without external pre-trained weights. Input images were resized to 224×224 pixels. The final DE-HVT configuration used an embedding dimension of 192, three attention heads and six Transformer encoder blocks. The embedding dimension and number of attention heads were based on the DeiT-Tiny configuration (Touvron et al., 2021), whereas the network depth was determined through the ablation experiment described in Section 3.1.3.

The models were trained for 100 epochs with a batch size of 256. AdamW was used for optimisation, with an initial learning rate of $\;5\times 10^{-4}\;$and a weight decay of 0.05 (Loshchilov and Hutter, 2019). Following five warm-up epochs, the learning rate was reduced to $\;1\times 10^{-5}\;$using a cosine annealing schedule.

Data augmentation has also been shown to improve learning from plant-disease images (Douarre et al., 2019). RandAugment, Mixup, CutMix and Random Erasing were used for data augmentation and regularisation (Zhang et al., 2018; Yun et al., 2019; Cubuk et al., 2020; Zhong et al., 2020). Stochastic depth and exponential moving average were also applied during training (Polyak and Juditsky, 1992; Huang et al., 2016). All compared models were trained from scratch using the same data partitions, data augmentation strategy, optimiser, learning-rate schedule, batch size and number of training epochs. The validation subset was used during model development, and the reported classification results were calculated exclusively from the held-out test subset.

#### 2.6.2 Comparative evaluation

DE-HVT was compared with five representative lightweight Vision Transformer architectures: DeiT-Tiny (Touvron et al., 2021), MobileViT-XS (Mehta and Rastegari, 2022), EdgeViT-XS (Pan et al., 2022), PiT-Tiny (Heo et al., 2021) and DHVT-Tiny (Lu et al., 2025). The baseline set was selected according to three criteria: a standard Tiny or XS configuration within a comparable parameter range, the use of Transformer-based global modelling, and coverage of distinct lightweight architectural strategies rather than multiple variants from the same model family. DeiT-Tiny represents a compact conventional Vision Transformer, PiT-Tiny represents hierarchical token reduction, MobileViT-XS combines convolutional local processing with Transformer-based global modelling, EdgeViT-XS introduces edge-oriented local aggregation and sparse global attention, and DHVT-Tiny represents a recent dynamic hybrid design. The purpose of this comparison was therefore to determine whether the proposed dual-embedding and local–global–local processing strategy provides a stable recognition–efficiency trade-off relative to closely related lightweight token-based architectures, rather than to construct an exhaustive leaderboard across all lightweight vision-model families.

The standard Tiny or XS architectural configurations described in the corresponding studies were retained for the baseline models, apart from replacing the final classification head to match the number of categories in each dataset. To isolate architectural differences, all models were evaluated on the same Plant Pathology and PlantVillage test subsets using the 224 × 224, from-scratch training settings described in Section 2.6.1. Each comparative experiment was repeated in three independent runs, and the results are reported as mean ± standard deviation. Parameter counts and FLOPs were calculated using the same procedure at an input resolution of 224 × 224. Top-1 accuracy and macro-averaged precision, recall and F1 score were used to assess classification performance, while confusion matrices were generated to examine class-specific prediction behaviour.

#### 2.6.3 Ablation experiments

Ablation experiments were conducted on the Plant Pathology dataset to determine the individual and combined contributions of OBPE, TexPE and ConvFFN. The baseline model used conventional non-overlapping patch embedding and a standard feed-forward network. OBPE and TexPE were then introduced individually and jointly, followed by the replacement of the conventional feed-forward network with ConvFFN. Only the component under investigation was changed between the corresponding configurations, while the remaining training and evaluation settings were kept unchanged. The classification metrics reported in **Tables 2**, **3** and **5** were obtained from a single run for each configuration.

**Table 2:** Ablation of Core Architecture.

Model	PE	TexPE	OBPE	FFN	ConvFFN	Param (M)	FLOPs (G)	Latency (ms)	Accuracy (%)	F1-score(%)
M1	√			√		3.92	0.64	0.07	85.10	84.95
M2		√		√		3.92	0.81	0.09	89.03	89.10
M3			√	√		3.92	0.54	0.06	85.55	85.34
M4		√	√	√		3.92	0.84	0.10	89.91	90.02
M5	√				√	4.10	0.65	0.07	91.29	91.33
M6		√			√	4.10	0.82	0.09	92.07	91.98
M7			√		√	4.10	0.56	0.06	90.32	90.45
M8		√	√		√	4.10	0.85	0.10	93.48	93.67

**Table 3:** Joint sensitivity of model performance and efficiency to patch-window size and overlap under a fixed 16-pixel stride.

Model	Window size	Overlap	Tokens	Param (M)	FLOPs(G)	Latency (ms)	Accuracy (%)
M8-Base	16	0	196	3.98	0.96	0.16	92.93
M8-1	17	1	169	4.10	0.85	0.10	93.48
M8-2	18	2	169	4.23	0.89	0.12	91.60
M8-3	19	3	169	4.36	0.93	0.14	91.84

A second ablation experiment examined the influence of the patch-window and overlap configurations. The configurations reported in **Table 3** were evaluated to determine how overlapping patch partitioning affected recognition performance, parameter count, FLOPs and inference latency. The associated sequence lengths are reported in the ablation results rather than in the architectural description.

A third ablation experiment examined the influence of network depth. DE-HVT variants containing 3, 6, 9 and 12 Transformer encoder blocks were evaluated using the same training protocol. This experiment was designed to determine whether the proposed dual-branch embedding and ConvFFN reduced the dependence of the model on a deep Transformer backbone.

#### 2.6.4 Frequency-domain and qualitative analyses

##### 2.6.4.1 Frequency-domain analysis

A two-dimensional Fast Fourier Transform (2D FFT) was applied to the spatial feature maps extracted from the relevant stages of the compared models. The zero-frequency component was shifted to the centre of the spectrum. In this representation, the central region mainly reflects slowly varying structural information, whereas peripheral components are associated with finer spatial variations, such as edges and textures. This interpretation is also consistent with the spectral bias commonly reported in neural networks, which describes the tendency to learn low-frequency components more readily than high-frequency components (Rahaman et al., 2019).

For reproducibility, the spectral analysis followed a fixed processing pipeline at every selected stage. The token sequence of shape (B, N, C) was first reshaped into a two-dimensional feature map with H = W = √N according to its spatial arrangement (13 × 13 when N = 169). The feature map was resized to 224 × 224 by bilinear interpolation and averaged across channels to obtain a single-channel map. The two-dimensional FFT was then computed, the zero-frequency component was shifted to the centre, and the absolute Fourier magnitude was used for the subsequent masking and proportion calculations.

An ideal circular mask was used to separate the low-frequency region from the remaining mid- and high-frequency region:

(24) $${\text{M}}_{\text{low}}(\text{u},\text{v})\;=\{\begin{array}{c} 1,{\left(u-{\text{u}}_{\text{c}}\right)}^{2}+{\left(v-{\text{v}}_{\text{c}}\right)}^{2}\leq {\text{r}}^{2}\\ 0,\text{otherwise} \end{array}$$

where $\;({\text{u}}_{\text{c}},{\text{v}}_{\text{c}})\;$denotes the centre of the shifted spectrum and r is the radius of the low-frequency region. The primary cutoff radius was set to r=20, which was selected as a moderate central-frequency threshold for the 224×224 Fourier representation. It retains the dominant slowly varying components while excluding most peripheral components associated with finer spatial variations. This value was selected for analysis convenience rather than because it represents a biologically determined frequency boundary. To assess the sensitivity of the results to the cutoff selection, additional analyses were conducted with r=15 and r=25, while all other processing conditions were kept unchanged. Let  F(u,v) denote the shifted Fourier spectrum. Because the analysis used the absolute Fourier magnitude, the measured quantity is reported as a relative spectral-magnitude proportion rather than a physical energy ratio:

(25) $${\text{R}}_{\text{low}}=\frac{\sum_{\text{u},\text{v}}{\text{M}}_{\text{low}}(\text{u},\text{v})|F(\text{u},\text{v})|}{\sum_{\text{u},\text{v}}|F(\text{u},\text{v})|}$$

(26) $${\text{R}}_{\text{mid}-\text{high}}=\;1-{\text{R}}_{\text{low}}$$

**Equations 24**–**26** define the circular low-frequency mask and the corresponding low-frequency and combined mid- and high-frequency magnitude proportions. The percentages reported in **Table 4** are calculated as $100\times R_{\text{mid}-\text{high}}$. Specifically, the numerator is the sum of the absolute Fourier magnitudes outside the circular low-frequency region, and the denominator is the sum over the entire spectrum. The cutoff radius *r* is measured in frequency-grid bins. Here, “mid- and high-frequency” denotes the combined region outside the low-frequency circle. The metric describes the relative spectral-magnitude distribution of the analysed features; it does not represent an energy ratio or the percentage of input-image information retained.

**Table 4:** Sensitivity of the measured mid- and high-frequency magnitude proportions to the circular cutoff radius.

Feature representation	r = 15	r = 20	r = 25
TexPE	98.2%	97.6%	86.2%
OBPE	29.3%	25.6%	22.8%
Fused embedding	51.2%	44.7%	39.5%
MHSA sublayer output	2.4%	2.1%	1.9%
ConvFFN sublayer output	63.8%	55.3%	48.6%

The analysis compared the feature distributions produced by conventional patch embedding, OBPE and TexPE during the initial representation stage. At the feature-transformation stage, the quantitative analysis compared the representations before and after the standard FFN in DeiT with those before and after ConvFFN in DE-HVT. The corresponding relative spectral-magnitude proportions are reported in **Figure 6**. DWConvFFN was included only in the qualitative log-magnitude spectrum comparison in **Figure 7**. These comparisons were used to examine whether TexPE introduces additional local-detail responses at the embedding stage and whether ConvFFN complements the globally modelled representation with locally filtered features.

**Figure 6:**
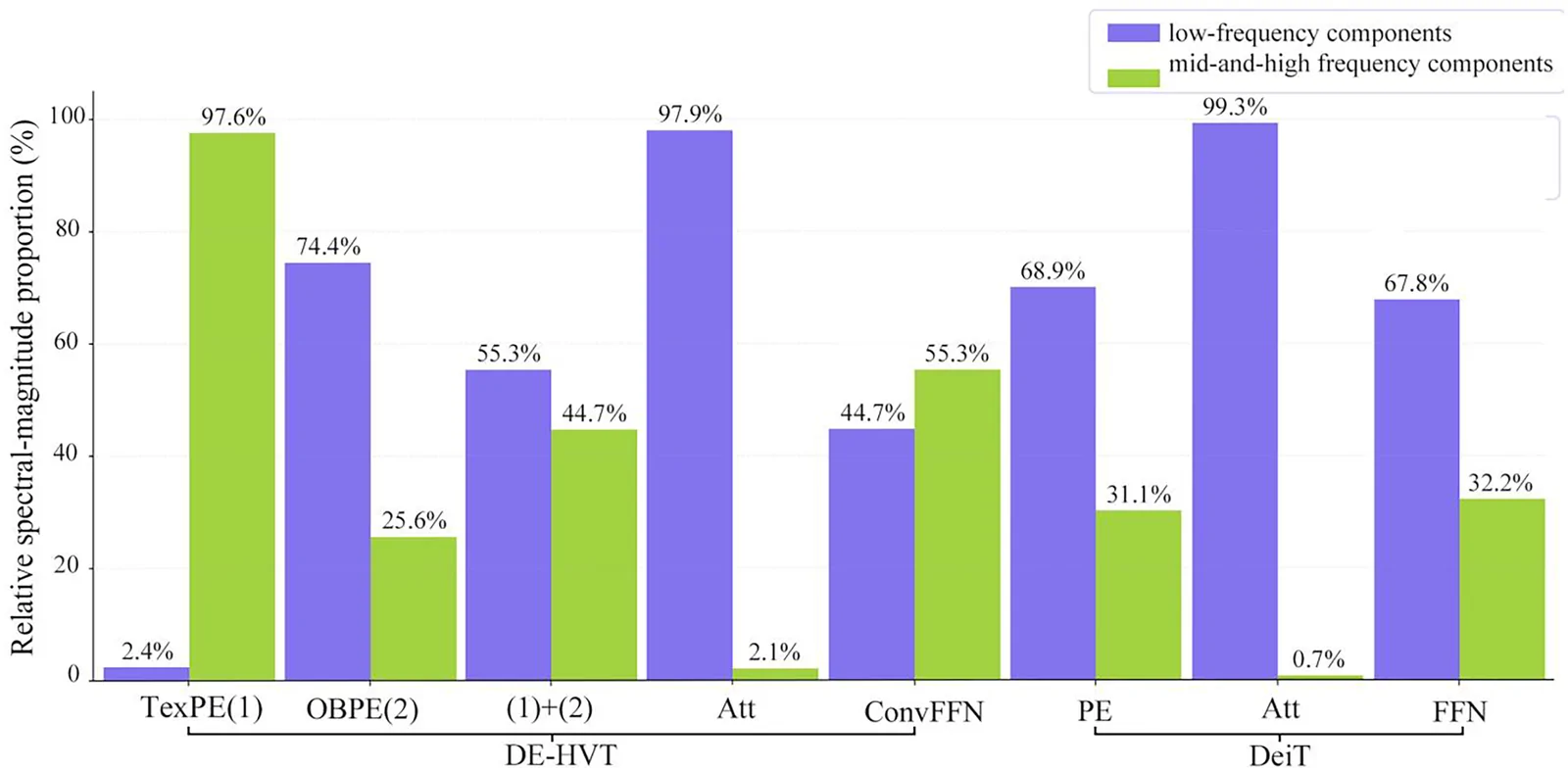
Relative spectral-magnitude proportions of intermediate feature representations in the compared feature-modelling pathways. The blue and green bars represent the proportions of low-frequency and mid- and high-frequency components, respectively. For DE-HVT, the plot shows the representations produced by TexPE, OBPE, their fused embedding, the MHSA sublayer and the subsequent ConvFFN sublayer. For DeiT, the plot shows the representations produced by conventional patch embedding, the MHSA sublayer and the subsequent standard FFN sublayer. The reported proportions are used to characterise the relative spatial-frequency biases of the intermediate representations and do not represent an explicit decomposition performed during model inference.

**Figure 7:**
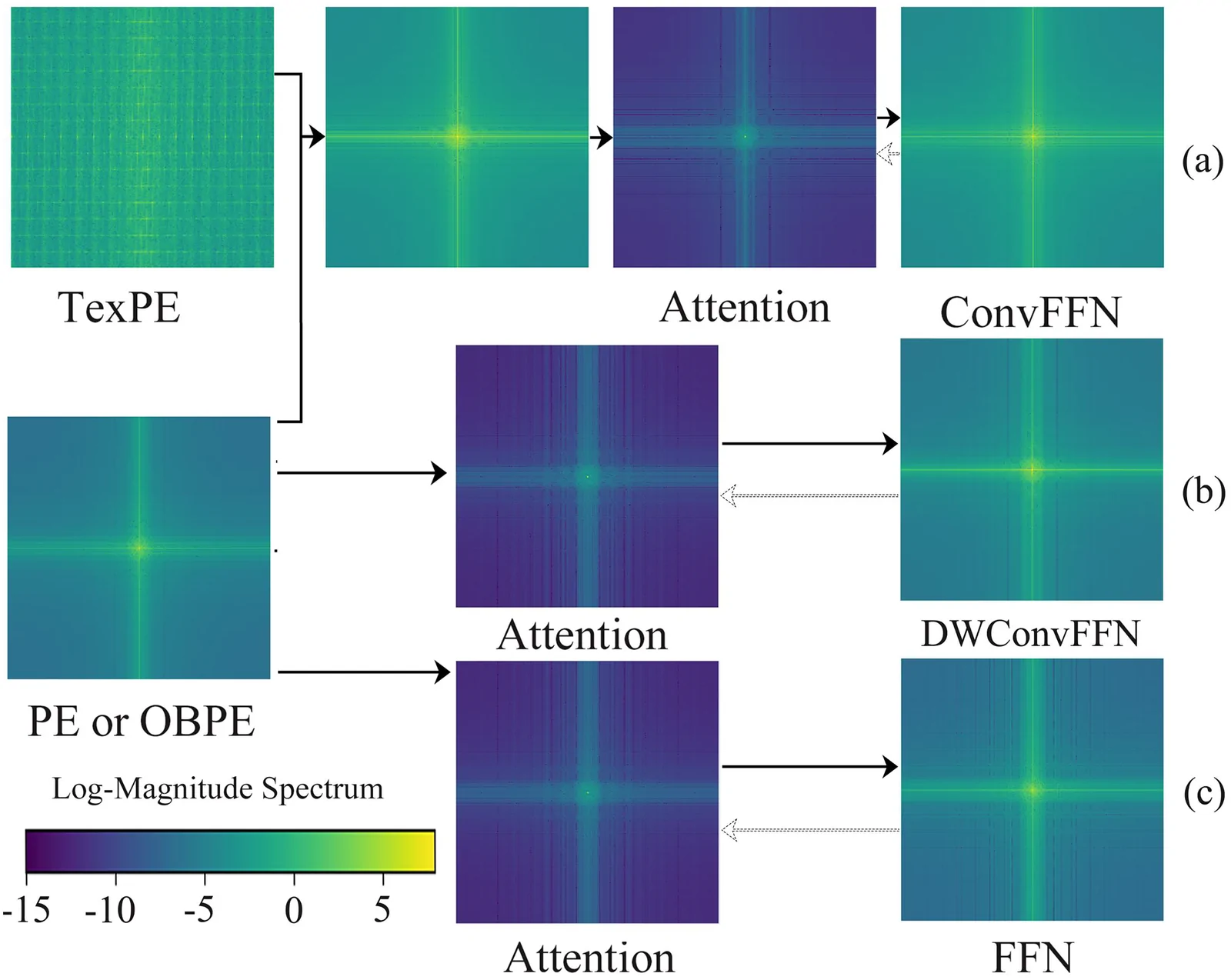
Log-magnitude spectra of feature representations from different feature-modelling pathways. The central region of each spectrum mainly represents slowly varying structural information, whereas the peripheral region reflects finer spatial variations associated with edges and textures. **(a)** TexPE followed by ConvFFN; **(b)** conventional Patch Embedding or OBPE followed by DWConvFFN; and **(c)** conventional Patch Embedding or OBPE followed by the standard FFN.

##### 2.6.4.2 Attention-map visualisation

To qualitatively examine the spatial distribution of the model response, attention weights were extracted from the final MHSA block of DE-HVT and the compared Transformer-based models. The attention weights were averaged across attention heads, reshaped according to the spatial arrangement of the embedded regions and upsampled to the input-image resolution. The resulting heatmaps are presented in **Figure 8**.

**Figure 8:**
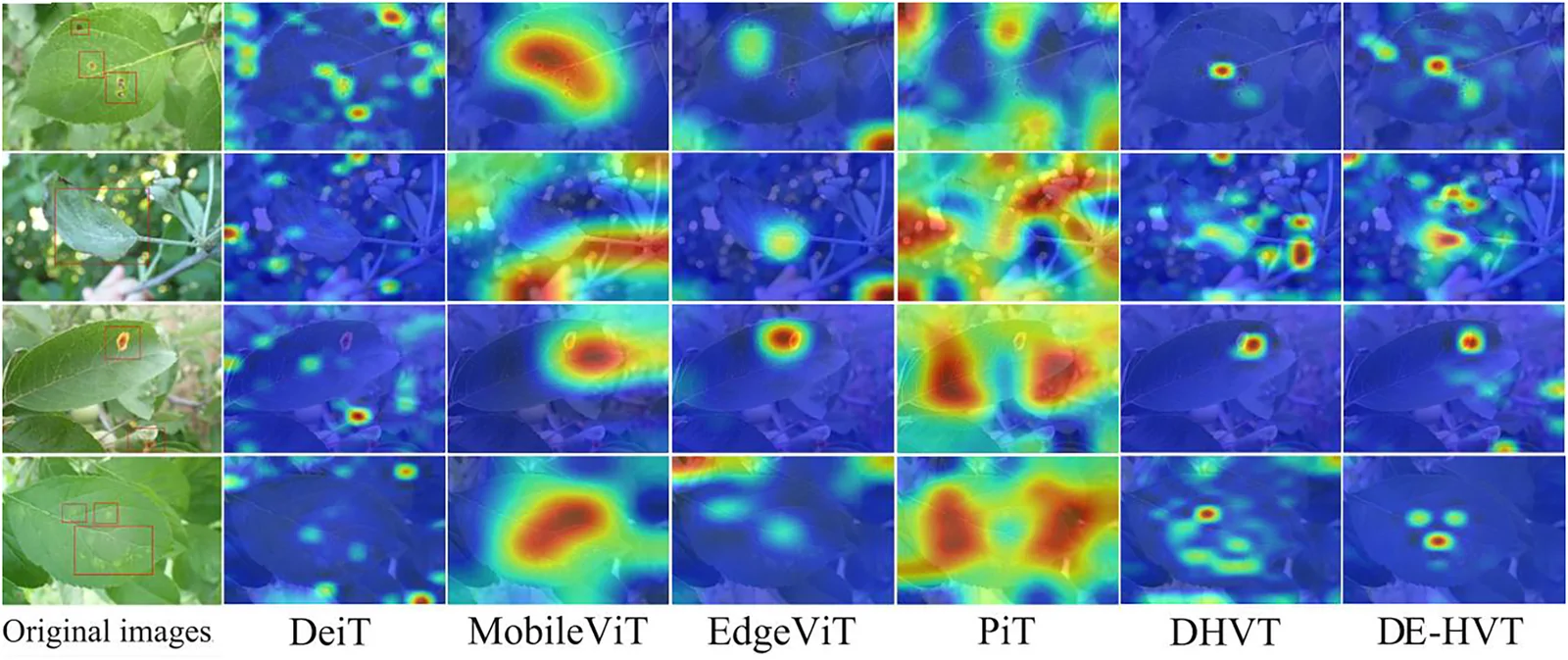
Qualitative visualisation of final-layer attention responses for representative Plant Pathology images. One representative test image was selected for each disease category included in the visualisation. The columns show the original images and the attention maps generated by DeiT-Tiny, MobileViT-XS, EdgeViT-XS, PiT-Tiny, DHVT-Tiny and DE-HVT, respectively. Warmer colours indicate higher attention responses, while the red boxes in the original images indicate visually apparent disease symptoms. The heatmaps illustrate the spatial distribution of model responses and should not be interpreted as lesion segmentation maps or quantitative localisation results.

The visualisations were used to qualitatively inspect model-response distributions in selected representative cases, including images with small lesions, complex orchard backgrounds and visually similar disease symptoms. The heatmaps were interpreted as qualitative evidence of the learned attention-response distributions.

#### 2.6.5 Mobile-device evaluation

On-device evaluation was conducted on a Redmi K30 Pro equipped with a Qualcomm Snapdragon 865 processor. The models were executed using ONNX Runtime in the AidLux environment with single-threaded CPU inference. All compared models used the same input resolution and runtime configuration.

The test image was first converted into a 224 × 224 FP32 input tensor. For each model, 10 warm-up inferences were performed, followed by 100 consecutive synchronous inference runs. During each measurement, the timer was started immediately before the ONNX Runtime session.run() call and stopped immediately after the call returned the raw model output. The maximum latency observed across the 100 measured runs was reported. Peak memory consumption was monitored during the same measurement process. The reported FPS was calculated as 1000 divided by the maximum observed latency in milliseconds. It therefore represents an FPS equivalent derived from the maximum observed latency rather than the typical inference rate. Mean and median latency are not reported in the current evaluation. All reported values were rounded to two decimal places.

#### 2.6.6 Evaluation metrics

For a classification task containing C classes, Top-1 accuracy was calculated as:

(27) $$\text{Accuracy}=\frac{\sum_{\text{i}=1}^{\text{C}}{\text{TP}}_{\text{i}}}{{\text{N}}_{\text{total}}}$$

where ${\text{ TP}}_{\text{i}}\;$is the number of correctly classified samples from class i, and ${\text{ N}}_{\text{total}}\;$is the total number of test samples.

Macro-averaged precision and recall were calculated as:

(28) $${\text{Precision}}_{\text{macro}}=\;\frac{1}{\text{C}}\sum_{\text{i}=1}^{\text{C}}\frac{{\text{TP}}_{\text{i}}}{{\text{TP}}_{\text{i}}+{\text{FP}}_{\text{i}}}$$

(29) $${\text{Recall}}_{\text{macro}}=\;\frac{1}{\text{C}}\sum_{\text{i}=1}^{\text{C}}\frac{{\text{TP}}_{\text{i}}}{{\text{TP}}_{\text{i}}+{\text{FN}}_{\text{i}}}$$

where ${\text{ FP}}_{\text{i}}\;$and ${\text{ FN}}_{\text{i}}\;$denote the numbers of false-positive and false-negative predictions for class i, respectively. **Equations 27**–**29** define Top-1 accuracy, macro-averaged precision, and macro-averaged recall, respectively.

Macro-averaged F1 score was calculated by averaging the class-specific F1 scores:

(30) $$\text{F}1_{\text{macro}}=\;\frac{1}{\text{C}}\sum_{\text{i}=1}^{\text{C}}\frac{2{\text{P}}_{\text{i}}{\text{R}}_{\text{i}}}{{\text{P}}_{\text{i}}+{\text{R}}_{\text{i}}}$$

where ${\text{ P}}_{\text{i}}\;$and ${\text{ R}}_{\text{i}}\;$are the precision and recall of class i. Macro-averaging assigns the same importance to each class and is therefore less affected by differences in class frequency than overall accuracy. **Equation 30** defines the macro-averaged F1 score used in this study.

## 3 Results

### 3.1 Ablation results

The following analyses report the effects of the core architectural components, patch-window and overlap configuration, and encoder depth on the Plant Pathology dataset.

#### 3.1.1 Contribution of the core architectural components

**Table 2** presents the component-level ablation results. The baseline model, M1, used conventional Patch Embedding and the standard feed-forward network, achieving a Top-1 accuracy of 85.10% and an F1-score of 84.95%, with 3.92 million parameters, 0.64 GFLOPs and an inference latency of 0.07 ms.

Introducing TexPE in M2 increased the accuracy from 85.10% to 89.03% and the F1-score from 84.95% to 89.10%, corresponding to improvements of 3.93 and 4.15 percentage points, respectively. The parameter count remained unchanged at 3.92 million, while FLOPs increased from 0.64 to 0.81 GFLOPs and latency increased from 0.07 to 0.09 ms. These results indicate that the CDC-based texture branch provides useful discriminative information during the initial embedding stage, with a moderate increase in computational cost.

Replacing conventional Patch Embedding with OBPE in M3 increased the accuracy from 85.10% to 85.55% and the F1-score from 84.95% to 85.34%. Meanwhile, FLOPs decreased from 0.64 to 0.54 GFLOPs and latency decreased from 0.07 to 0.06 ms, while the parameter count remained unchanged. Under the evaluated configuration, OBPE therefore contributed mainly to computational efficiency and provided a limited independent improvement in recognition performance.

When TexPE and OBPE were combined in M4, the accuracy and F1-score increased to 89.91% and 90.02%, respectively. Compared with M1, these values represent improvements of 4.81 and 5.07 percentage points. Compared with M2, the corresponding improvements were 0.88 and 0.92 percentage points. M4 contained 3.92 million parameters, required 0.84 GFLOPs and recorded a latency of 0.10 ms. These results indicate that TexPE and OBPE provide complementary representations.

ConvFFN improved both accuracy and F1-score in every corresponding embedding configuration. Relative to M1–M4, the corresponding M5–M8 variants improved accuracy by 3.04–6.19 percentage points and F1-score by 2.88–6.38 percentage points. In each paired comparison, the parameter count increased by 0.18 million and FLOPs increased by only 0.01–0.02 GFLOPs. The reported latency remained unchanged within the two-decimal measurement precision: 0.07 ms for M1/M5, 0.09 ms for M2/M6, 0.06 ms for M3/M7 and 0.10 ms for M4/M8.

The complete model, M8, achieved the highest accuracy of 93.48% and the highest F1-score of 93.67%, with 4.10 million parameters, 0.85 GFLOPs and an inference latency of 0.10 ms. Compared with M4, adding ConvFFN improved accuracy and F1-score by 3.57 and 3.65 percentage points, respectively, while increasing the parameter count by 0.18 million and FLOPs by 0.01 GFLOPs, without increasing the reported latency. These results support the intended division of functions within DE-HVT: TexPE strengthens fine-grained initial representations, OBPE contributes to computational efficiency, and ConvFFN reinforces local spatial interactions after global feature modelling.

#### 3.1.2 A joint sensitivity analysis of window size and overlap

**Table 3** shows the effects of different configurations of window size and overlap. The latency values were measured using the protocol described in Section 2.6.1. The non-overlapping M8-Base configuration used a window size of 16 and zero overlap, producing 196 embedded regions. It achieved an accuracy of 92.93% with 3.98 million parameters, 0.96 GFLOPs and an inference latency of 0.16 ms.

When the window size was increased to 17 with an overlap of 1 in M8-1, the sliding stride remained 16. Because the convolution was applied without padding, the resulting spatial grid decreased from 14×14 to  13×13, reducing the sequence length from 196 to 169. This configuration reduced the computational cost from 0.96 to 0.85 GFLOPs and the reported inference latency from 0.16 to 0.10 ms. At the same time, accuracy increased by 0.55 percentage points, reaching 93.48%.

Further increases in both window size and overlap did not produce additional improvements. M8-2, with a window size of 18 and an overlap of 2, achieved an accuracy of 91.60%, while M8-3, with a window size of 19 and an overlap of 3, achieved 91.84%. Although both configurations maintained the same sequence length of 169, their parameter counts and computational costs increased because of the larger convolutional kernels. Their inference latencies also increased to 0.12 and 0.14 ms, respectively.

Under the evaluated settings, M8-1 (b=17,o=1) achieved the highest observed accuracy, with lower FLOPs and latency reported in **Table 3** than the non-overlapping M8-Base reference, although its parameter count was slightly higher. Relative to M8-1, further increases in window size and overlap in M8–2 and M8–3 increased the parameter count, FLOPs and latency, but resulted in lower accuracy. Accordingly, M8–1 was used as the final window–overlap configuration.

#### 3.1.3 Effect of network depth

The influence of network depth is reported in **Table 5**. The evaluated DE-HVT variants contained 3, 6, 9 or 12 Transformer encoder blocks. The six-block model achieved the highest values across all reported classification metrics, with an accuracy of 93.48%, macro-averaged precision of 93.81%, macro-averaged recall of 93.58% and macro-averaged F1 score of 93.67%.

**Table 5:** Impact of network depth on performance and efficiency.

Model	Depth	Param (M)	FLOPs(G)	Accuracy (%)	Precision(%)	Recall(%)	F1-score(%)
DeiT	12	6.59	1.25	84.54	86.43	84.19	85.10
M8-12	12	6.95	1.38	92.55	93.08	92.29	92.64
M8-9	9	5.53	1.32	92.12	93.28	92.03	92.58
M8-6	6	4.10	0.85	93.48	93.81	93.58	93.67
M8-3	3	2.67	0.59	91.42	92.86	90.55	91.61

Increasing the depth from six to nine blocks increased the parameter count from 4.10 to 5.53 million and the computational cost from 0.85 to 1.32 GFLOPs. However, accuracy decreased from 93.48% to 92.12%, while the macro-averaged F1 score decreased from 93.67% to 92.58%. Increasing the depth further to 12 blocks resulted in an accuracy of 92.55% and a macro-averaged F1 score of 92.64%, with 6.95 million parameters and 1.38 GFLOPs. Thus, additional encoder blocks did not provide a corresponding improvement in recognition performance under the evaluated training conditions.

Compared with the 12-block DE-HVT variant, the six-block model reduced the parameter count by approximately 41.0% and the computational cost by approximately 38.4%, while increasing accuracy by 0.93 percentage points. This result suggests that the complementary representations generated by the dual-branch embedding and ConvFFN reduce the need for a deep Transformer backbone. However, reducing the depth to three blocks lowered accuracy to 91.42% and the macro-averaged F1 score to 91.61%, indicating that excessive depth reduction limits representation capacity.

All DE-HVT depth variants outperformed the 12-block DeiT baseline in accuracy and macro-averaged F1 score. Notably, even the three-block DE-HVT achieved an accuracy of 91.42% with 2.67 million parameters and 0.59 GFLOPs, compared with 84.54%, 6.59 million parameters and 1.25 GFLOPs for DeiT. These results indicate that the performance improvement was not dependent solely on increasing network depth.

Accordingly, the six-block configuration was selected for the final DE-HVT model because it achieved the highest accuracy and F1-score among the evaluated depths while requiring fewer parameters and FLOPs than the deeper variants.

### 3.2 Frequency-domain analysis

#### 3.2.1 Sensitivity analysis of the frequency cutoff radius

To evaluate the sensitivity of the spectral measurements to the cutoff selection, the analysis was repeated using circular cutoff radii r∈{15,20,25}. All other processing conditions, including the input preprocessing, feature extraction locations, feature resizing, channel averaging and Fourier-magnitude calculation, were kept unchanged. The measured mid- and high-frequency proportions decreased as the cutoff radius increased because a larger central region was classified as low frequency. However, the relative ordering of the feature representations remained stable. TexPE consistently exhibited the highest mid- and high-frequency proportion, whereas the MHSA sublayer output showed the lowest. The ConvFFN sublayer output remained more responsive to spatially varying components than the MHSA output across all three cutoff values. These results indicate that the qualitative frequency-aware interpretation was not dependent on the specific choice of r=20. The complete results are summarised in **Table 4**.

#### 3.2.2 Main spectral analysis

Frequency-domain analysis was conducted to examine whether DE-HVT preserved local spatial details during feature transformation. The analysis focused on two stages of the network: the initial patch-embedding stage and the feature-transformation stage following global self-attention. The results are presented in **Figure 6**, while the corresponding log-magnitude spectra are visualised in **Figure 7**. Because the analysis was based on the absolute Fourier magnitude, the reported values represent relative spectral-magnitude proportions rather than physical energy ratios.

Initial feature representation. The spectral distributions of the initial representations are shown in **Figure 6**. The conventional Patch Embedding and OBPE representations were dominated by low-frequency components. The low-frequency proportion was 68.9% for conventional Patch Embedding and 74.4% for OBPE, whereas the corresponding mid- and high-frequency proportions were 31.1% and 25.6%, respectively. This distribution indicates that both branches mainly retained slowly varying structural information, such as the overall leaf contour and broad colour patterns, while providing a relatively smaller proportion of fine spatial variations.

In contrast, TexPE exhibited a higher relative mid- and high-frequency magnitude proportion than OBPE across the evaluated cutoff radii. At r=20, 97.6% of the total Fourier magnitude of the analysed TexPE representation lay outside the circular low-frequency region, while 2.4% lay inside it. This value describes the spectral distribution under the specified feature-processing and cutoff settings; it does not mean that TexPE extracts 97.6% of the input image’s high-frequency information or explicitly separates frequency bands. The observed distribution is consistent with a difference-sensitive representation, while its interpretation remains specific to the analysed features and calculation procedure.

When the OBPE and TexPE representations were combined, the proportion of mid- and high-frequency components increased from 25.6% for OBPE alone to 44.7%. This indicates that the two branches provide complementary spectral characteristics. OBPE retains broader structural information, whereas TexPE contributes additional fine-grained responses that are not sufficiently represented by the structural branch alone.

Feature changes after global self-attention. The analysed representations after the MHSA stage remained primarily low-frequency dominated. In the DE-HVT pathway, the feature map before ConvFFN contained 97.9% low-frequency components and only 2.1% mid- and high-frequency components. A similar tendency was observed in the DeiT pathway before its conventional feed-forward network, where the low-frequency proportion reached 99.3% and the mid- and high-frequency proportion was 0.7%. These results indicate that, under the analysed conditions, global feature interaction was associated with a smoother spectral representation and a lower relative contribution from fine spatial variations.

This observation is consistent with the complementary design motivation of DE-HVT. MHSA is used to establish long-range relationships among the embedded image regions, but the resulting globally aggregated representation may contain fewer explicitly local responses. Therefore, a subsequent local feature-transformation operation is required to preserve information that may be weakened during global interaction.

Local feature refinement by ConvFFN. The spectral distributions after feature transformation are shown in **Figure 6**. In the DeiT pathway, the conventional feed-forward network reduced the low-frequency proportion from 99.3% to 67.8% and increased the mid- and high-frequency proportion from 0.7% to 32.2%. In the DE-HVT pathway, ConvFFN reduced the low-frequency proportion from 97.9% to 44.7% and increased the mid- and high-frequency proportion from 2.1% to 55.3%. Thus, the increase in the mid- and high-frequency proportion was 53.2 percentage points for DE-HVT, compared with 31.5 percentage points for the DeiT pathway.

The log-magnitude spectra in **Figure 7** provide a qualitative illustration of this difference. The conventional PE/OBPE–FFN pathway remains strongly concentrated around the centre of the spectrum, indicating a greater dominance of slowly varying components. The PE/OBPE–DWConvFFN pathway produces more peripheral responses, but these responses remain weaker than those of the complete ConvFFN pathway. The TexPE–ConvFFN pathway shows the clearest peripheral spectral response, indicating a stronger representation of fine spatial variations after the global modelling stage.

Overall, the results support a frequency-aware interpretation of DE-HVT. TexPE produces stronger responses to fine spatial variations during initial embedding, whereas ConvFFN reinforces locally varying responses after global token interaction. The accompanying changes in spectral composition are consistent with the classification ablation results and indicate complementary frequency-biased processing across the proposed modules. However, these observations do not demonstrate an exact or orthogonal separation of frequency components, nor do they establish that any particular frequency component causally determines classification accuracy.

### 3.3 Comparison with representative lightweight Vision Transformer models

**Table 6** compares DE-HVT with five representative lightweight Vision Transformer models on the Plant Pathology and PlantVillage datasets. On the Plant Pathology dataset, DE-HVT achieved the highest mean values across all reported classification metrics, with an accuracy of 93.51±0.44%, precision of 93.82±0.18%, recall of 93.55±0.55% and macro-averaged F1 score of 93.64±0.40%. Compared with DHVT-Tiny, DE-HVT improved the mean accuracy and macro-averaged F1 score by 1.05 and 1.28 percentage points, respectively. Within the evaluated lightweight Transformer baselines, DE-HVT therefore achieved the strongest recognition performance on the more challenging Plant Pathology dataset while maintaining moderate model complexity.

**Table 6:** Comparison of DE-HVT with representative lightweight Vision Transformer models.

Model	Dataset	Parameters (M)	FLOPs (G)	Accuracy (%)	Precision (%)	Recall (%)	F1-score (%)
DeiT-Tiny	Plant Pathology	6.59	1.25	84.57 ± 0.71	86.40 ± 0.65	84.22 ± 0.76	85.07 ± 0.70
	PlantVillage			99.81 ± 0.07	99.74 ± 0.07	99.77 ± 0.08	99.73 ± 0.07
MobileViT-XS	Plant Pathology	2.00	0.72	89.07 ± 0.68	89.90 ± 0.63	88.15 ± 0.75	88.99 ± 0.67
	PlantVillage			99.44 ± 0.09	99.27 ± 0.08	99.33 ± 0.09	99.31 ± 0.08
EdgeViT-XS	Plant Pathology	6.44	1.10	89.86 ± 0.66	90.10 ± 0.61	89.07 ± 0.72	89.52 ± 0.65
	PlantVillage			99.80 ± 0.08	99.74 ± 0.07	99.74 ± 0.08	99.72 ± 0.07
PiT-Tiny	Plant Pathology	4.98	0.7	83.79 ± 0.75	84.65 ± 0.70	83.64 ± 0.78	84.13 ± 0.73
	PlantVillage			99.12 ± 0.10	99.08 ± 0.09	98.95 ± 0.10	99.04 ± 0.09
DHVT-Tiny	Plant Pathology	5.99	1.38	92.46 ± 0.42	93.44 ± 0.18	91.43 ± 0.58	92.36 ± 0.42
	PlantVillage			99.57 ± 0.08	99.54 ± 0.07	99.50 ± 0.08	99.52 ± 0.07
DE-HVT(Ours)	Plant Pathology	4.10	0.85	93.51 ± 0.44	93.82 ± 0.18	93.55 ± 0.55	93.64 ± 0.40
	PlantVillage			99.82 ± 0.06	99.66 ± 0.07	99.77 ± 0.07	99.70 ± 0.07

The advantage of DE-HVT was most evident on Plant Pathology, where disease recognition is affected by natural backgrounds, variable illumination and subtle visual differences between symptoms. The results are consistent with the intended combination of texture-sensitive initial embedding, global contextual modelling and subsequent local feature refinement. However, the contributions of the individual components are evaluated separately in the ablation experiments rather than inferred solely from this comparison.

On PlantVillage, all models achieved very high recognition performance. DE-HVT obtained a mean accuracy of 99.82 ± 0.06%, compared with 99.81 ± 0.07% for DeiT-Tiny and 99.80 ± 0.08% for EdgeViT-XS. Its mean recall of 99.77 ± 0.07% was comparable to that of DeiT-Tiny, whereas its mean precision and macro-averaged F1 score were slightly lower than those of the strongest competing models. The near-ceiling performance is consistent with the relatively controlled acquisition conditions, clearly visible leaf regions and standardised image composition of PlantVillage. A more detailed interpretation of these dataset-related characteristics is provided in Section 4.1.

DE-HVT contained 4.10 million parameters and required 0.85 GFLOPs. Compared with DHVT-Tiny, it reduced the parameter count by approximately 31.6% and FLOPs by approximately 38.4%. It also required fewer parameters and FLOPs than DeiT-Tiny and EdgeViT-XS. However, DE-HVT did not achieve the lowest model complexity among all evaluated architectures: MobileViT-XS contained fewer parameters, while MobileViT-XS and PiT-Tiny required fewer FLOPs. Therefore, the advantage of DE-HVT lies in achieving higher Plant Pathology recognition performance with moderate model complexity rather than in minimising every individual complexity measure.

### 3.4 Class-wise classification results

Confusion matrices were analysed to examine the distribution of predictions across disease categories. The results for PlantVillage and Plant Pathology are presented in **Figure 9** and **Figure 10**, respectively.

**Figure 9:**
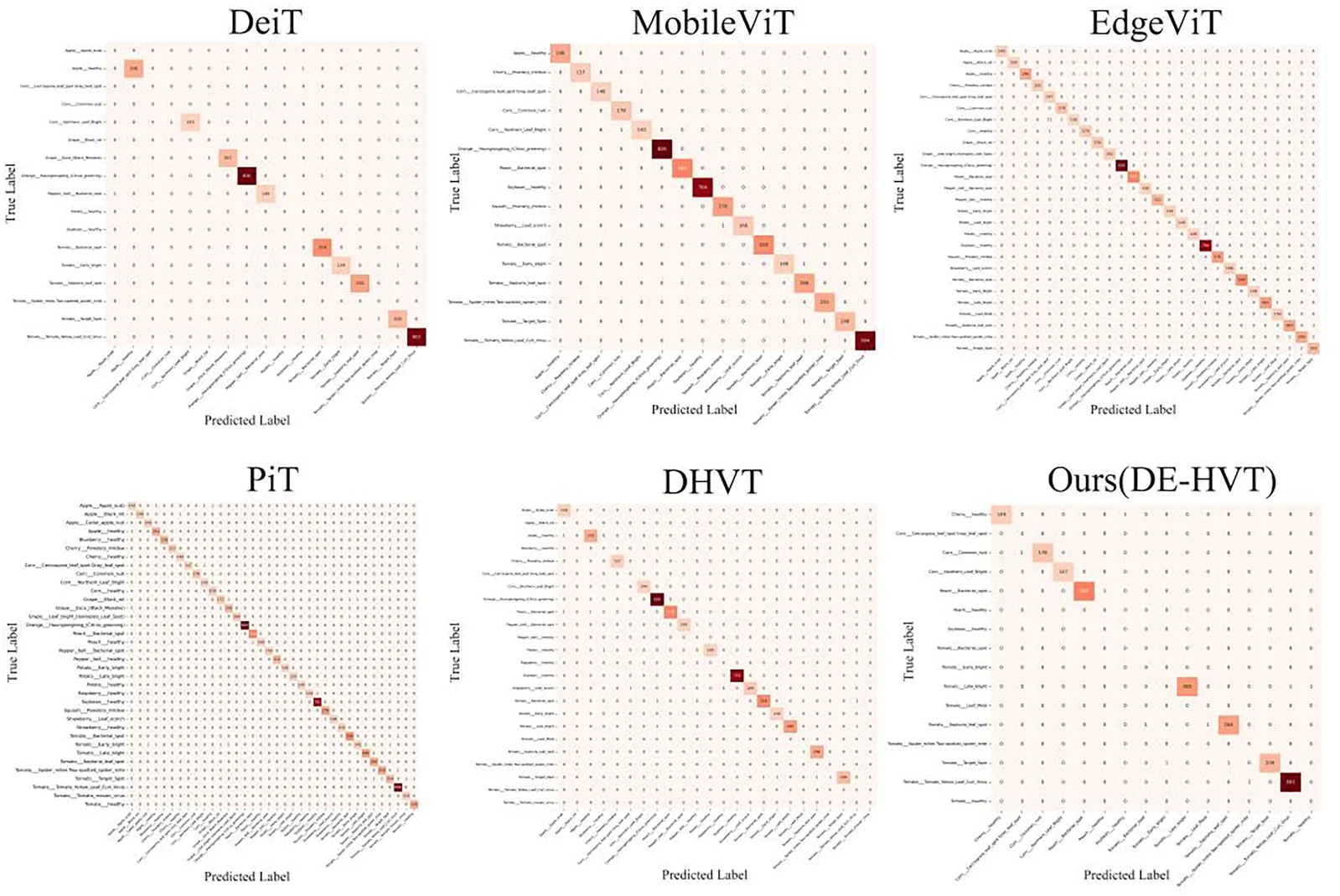
Confusion matrices for the selected lightweight models on the PlantVillage test set. Rows represent the true class labels, columns represent the predicted class labels, and each cell reports the number of test images assigned to the corresponding true–predicted class pair.

**Figure 10:**
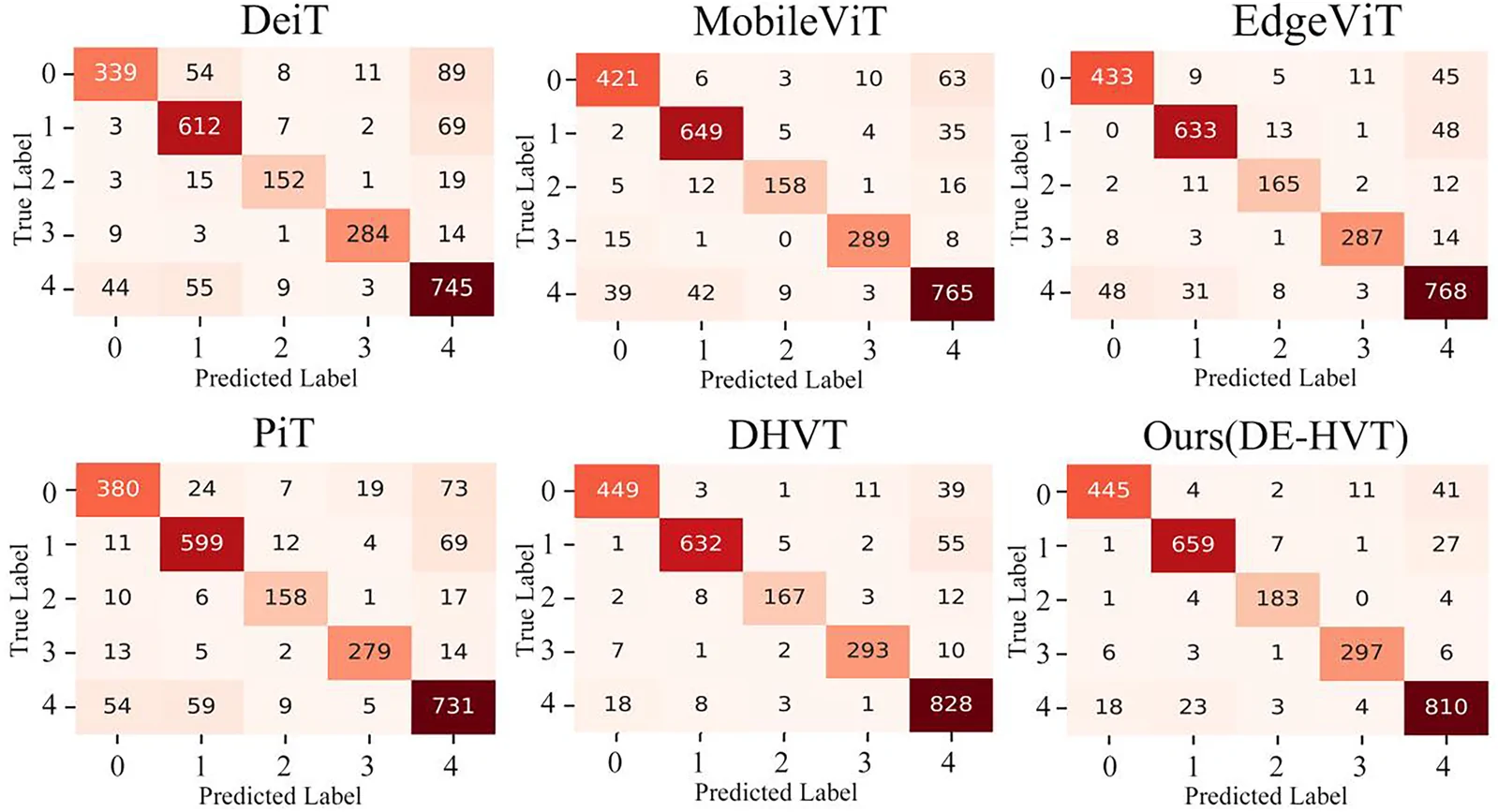
Confusion matrices for the selected lightweight models on the Plant Pathology test set. Rows represent the true class labels and columns represent the predicted class labels. Class labels 0–4 correspond to frogeye leaf spot, healthy, powdery mildew, rust and apple scab, respectively. Each cell reports the number of test images assigned to the corresponding true–predicted class pair.

On PlantVillage, the confusion matrices of all compared models were strongly concentrated along the main diagonal, consistent with the high classification scores reported in **Table 6**. The limited off-diagonal predictions indicate that most categories were readily distinguished under the controlled imaging conditions. However, the matrices do not provide sufficient evidence to conclude that DE-HVT was uniformly more robust than all comparison models on this dataset.

More pronounced class-wise differences were observed on Plant Pathology. In **Figure 10**, class labels 0–4 correspond to frogeye leaf spot, healthy, powdery mildew, rust and apple scab, respectively. Within the DE-HVT results, frogeye leaf spot yielded the lowest recall at 88.47%, while the other four categories achieved recalls above 94%. The largest pairwise confusion involved frogeye leaf spot and apple scab: 41 frogeye leaf spot images were assigned to apple scab, while 18 apple scab images were assigned to frogeye leaf spot.

The comparison with DHVT-Tiny revealed a class-specific precision–recall trade-off for apple scab. DE-HVT correctly classified fewer apple scab images than DHVT-Tiny (810 versus 828) and produced more false negatives (48 versus 30). However, DE-HVT produced fewer false-positive apple scab predictions (78 versus 116). Consequently, DE-HVT achieved higher precision for apple scab (91.22% versus 87.71%) but lower recall (94.41% versus 96.50%).

Overall, DE-HVT produced 167 misclassifications on the Plant Pathology test set, 25 fewer than DHVT-Tiny, which produced 192. This reduction was associated mainly with improved classification of healthy leaves, powdery mildew and rust, although DHVT-Tiny retained a higher recall for apple scab. The advantage of DE-HVT was therefore evident at the overall class-wise level but was not uniform across all disease categories. The remaining errors were concentrated primarily between frogeye leaf spot and apple scab under the evaluated test conditions.

### 3.5 Qualitative attention visualisation

**Figure 8** provides an illustrative qualitative comparison of the spatial distributions of model responses on selected Plant Pathology test images. One representative image was selected for each disease category included in the visualisation. The attention maps generated by PiT-Tiny, MobileViT-XS, DeiT-Tiny, EdgeViT-XS, DHVT-Tiny and DE-HVT are shown for visual comparison. Warmer colours indicate regions associated with higher attention responses, whereas the red boxes in the original images mark visually apparent disease symptoms.

In the displayed examples, PiT-Tiny and MobileViT-XS showed relatively broad response regions across parts of the leaves and, in some cases, over nearby background areas. DeiT-Tiny produced more spatially separated responses in several examples, although some high-response regions were also located outside the visually apparent symptom areas. These observations describe the response patterns in the selected images and are not intended to represent the general behaviour of these models across the dataset.

By comparison, DHVT-Tiny and DE-HVT showed relatively concentrated response regions. In some images containing isolated lesions, their stronger responses overlapped with the visually apparent symptom regions. In examples with multiple or dispersed symptoms, DE-HVT showed several local response peaks. These patterns provide illustrative information about the response distributions in the selected cases, but they do not establish a general advantage in lesion localization or background suppression.

The displayed examples are qualitatively consistent with the design objective of examining lesion-related responses under complex backgrounds. However, the analysis is based on one representative test image per disease category, and no pixel-level lesion annotations were available. The heatmaps are therefore used only to illustrate attention-response distributions in the selected cases and should not be interpreted as a quantitative assessment of lesion localisation, segmentation performance or dataset-wide model behaviour.

### 3.6 On-device performance

**Table 7** presents the on-device performance measured using the protocol described in Section 2.6.5.

**Table 7:** Mobile-platform performance of the compared lightweight Vision Transformer models.

Model	Mobile memory (M) ↓	Mobile latency (ms) ↓	FPS equivalent ↑
PiT-Tiny	68.59	49.7	20.12
EdgeViT-XS	48.41	63.17	15.83
DeiT-Tiny	49.29	82.21	12.16
MobileViT-XS	35.62	142.11	7.04
DHVT-Tiny	59.82	271.76	3.68
DE-HVT	46.86	111.96	8.93

Under the tested device and runtime configuration, DE-HVT required 46.86 MB of peak memory and recorded a maximum observed latency of 111.96 ms per image, corresponding to an FPS equivalent of 8.93. Its peak memory consumption was 31.68% lower than that of PiT-Tiny, 4.93% lower than that of DeiT-Tiny, 3.20% lower than that of EdgeViT-XS and 21.67% lower than that of DHVT-Tiny. Among the evaluated models, only MobileViT-XS used less memory, with a peak consumption of 35.62 MB.

The maximum observed latency of DE-HVT was 159.80 ms lower than that of DHVT-Tiny and 30.15 ms lower than that of MobileViT-XS. However, it was higher than those of DeiT-Tiny, PiT-Tiny and EdgeViT-XS, which recorded maximum observed latencies of 82.21, 49.70 and 63.17 ms per image, respectively.

Overall, DE-HVT exhibited an intermediate device-level efficiency profile under the tested mobile conditions. Although it did not achieve the lowest maximum observed latency, it required the second-lowest peak memory and remained feasible for mobile inference under the tested configuration. When the classification results in **Table 6** and the mobile measurements in **Table 7** are considered together, the principal advantage of DE-HVT is its higher Plant Pathology recognition performance with moderate memory consumption, rather than universal superiority in device-level speed.

## 4 Discussion

### 4.1 Overall performance and model effectiveness

The comparative results show that DE-HVT performed most strongly on the more challenging Plant Pathology dataset, whereas all evaluated models achieved near-ceiling recognition performance on PlantVillage.

The lower performance on Plant Pathology than on PlantVillage reflects the differences in imaging conditions between the two datasets. Plant Pathology contains complex natural backgrounds, variable illumination and greater variation in symptom appearance, whereas PlantVillage contains detached leaves photographed individually against grey or black background sheets under relatively controlled acquisition conditions (Mohanty et al., 2016; Barbedo, 2018). Its clearly visible leaf regions, readily observable disease characteristics, standardised image composition and limited background interference make the crop–disease categories easier to distinguish and contribute to the near-ceiling performance observed across the evaluated models. In addition, visual similarity among non-identical images, together with the adopted image-level partition, may have contributed to the high PlantVillage results. Consequently, the small numerical differences among the compared models provide limited practical separation on PlantVillage, whereas Plant Pathology offers a more informative setting for comparing model robustness under background interference, illumination changes and subtle disease symptoms.

The PlantVillage evaluation used an image-level stratified split, which does not ensure that images of the same physical leaf are confined to a single subset. Images from the same leaf may therefore occur in both the training and test subsets. Accordingly, the very high PlantVillage results, including the 99.82 ± 0.06% accuracy achieved by DE-HVT, should be interpreted as performance under the current benchmark split and may not fully reflect performance on completely independent data.

### 4.2 Contribution of the DE-HVT architecture

The results support the complementary roles of the four principal components of DE-HVT. OBPE preserves overlapping spatial information between neighbouring patches, while TexPE enhances local contrast, lesion boundaries and texture changes through central-difference convolution. By fusing these two representations before MHSA, the model can perform global feature modelling on token representations that already contain both structural and texture-related information.

The window–overlap analysis further suggests that the benefit of local contextual sharing depends on the joint configuration of window size and overlap. Because window size and overlap were varied jointly under a fixed stride, these results should not be interpreted as a monotonic effect of overlap alone. M8-Base serves as the non-overlapping reference, whereas the overlapping configurations maintain a fixed 16-pixel stride, thereby preserving the same non-shared spatial advance while progressively enlarging the shared contextual region. A narrow overlap may be sufficient to reduce discontinuities at patch boundaries, whereas larger shared regions may provide diminishing returns by increasing redundancy between neighbouring representations and incorporating additional background or illumination variation that is less relevant to lesion discrimination in the Plant Pathology images. The larger projection kernels also increase model complexity. Within the evaluated settings, these competing effects are consistent with M8–1 providing the most favourable balance among cross-patch continuity, recognition accuracy and computational complexity.

ConvFFN further complements self-attention by introducing local spatial processing after global feature interactions. This is important because self-attention is effective for modelling long-range relationships but does not explicitly provide convolutional local priors. The ablation results indicate that the combined use of OBPE, TexPE and ConvFFN is more effective than using any individual component alone. The frequency-domain analysis further shows that the proposed sublayers exhibit different but complementary spectral characteristics, and these relative trends remain stable across the evaluated cutoff values, supporting a frequency-aware interpretation of the architecture. Accordingly, the frequency-aware behaviour of DE-HVT should be understood as complementary and frequency-biased rather than as exact, orthogonal or lossless frequency separation. These spectral results provide supporting mechanistic evidence, but they do not establish a causal relationship between any particular frequency component and classification accuracy or specific biological symptoms.

Methods developed for fine-grained visual recognition commonly emphasise the localisation or representation of discriminative object parts and subtle local attributes (Wei et al., 2022). In plant disease recognition, Chang et al. (2024) introduced explicit edge-feature guidance to strengthen disease-boundary representations in Vision Transformers. In contrast, DE-HVT learns difference-sensitive representations through CDC during patch embedding and integrates them with spatially aligned structural tokens before self-attention. It therefore preserves local symptom information through the embedding design rather than through a separate edge-guidance mechanism.

The present study was designed to evaluate DE-HVT within a lightweight, edge-oriented setting. Accordingly, the numerical comparison in **Table 6** was restricted to Tiny/XS architectures containing 2.00–6.59 million parameters and evaluated under the same 224 × 224, from-scratch training protocol. A direct comparison with mainstream specialised FGVC models, such as TransFG (He et al., 2022) and E-FGVC (Chang et al., 2023), falls outside this design-focused comparison because their standard configurations contain approximately 86.85 million and 29.23–32.48 million parameters, respectively, and employ different backbone, pretraining or multi-dataset learning settings. A dedicated same-dataset benchmark for representative FGVC architectures will therefore be established in future work using the Plant Pathology and PlantVillage partitions, with recognition performance, parameter count, FLOPs and mobile-device inference latency jointly evaluated.

Beyond this FGVC-specific extension, the present comparison does not constitute an exhaustive benchmark across all lightweight vision-model families. Therefore, the current findings should not be interpreted as demonstrating universal superiority over all edge-oriented architectures. Future work will also extend the lightweight comparison to representative CNNs and efficient Transformer architectures, including MobileNet, EfficientNet, ShuffleNet, EfficientFormer and Mobile-Former.

### 4.3 Class-wise behaviour and mobile deployment

The confusion matrices provide additional evidence of the fine-grained nature of the Plant Pathology task. The remaining errors of DE-HVT were concentrated mainly between frogeye leaf spot and apple scab, for which some evaluated images exhibited visually similar local lesion appearances. Because the overall apple-leaf morphology is shared across these categories, their discrimination may depend on subtle differences in lesion shape, boundary definition, colour distribution and texture. This class-specific confusion is consistent with the small inter-class differences characteristic of fine-grained recognition. It also shows that the improvement of DE-HVT was not uniform across all disease categories and that visually overlapping symptom patterns remain challenging.

The mobile-platform comparison indicates that the additional texture-sensitive branch did not preclude practical mobile inference. Although DE-HVT did not achieve the lowest maximum observed latency, it combined the second-lowest peak memory consumption with the strongest Plant Pathology recognition performance among the evaluated lightweight models. Its deployment value therefore lies primarily in balancing recognition performance and memory efficiency rather than in achieving universal superiority in inference speed. This interpretation is consistent with the recent emphasis on lightweight and edge-oriented plant disease recognition (Zeeshan et al., 2025).

These deployment results are specific to the tested hardware and runtime configuration and demonstrate feasibility under the evaluated conditions rather than universal superiority across mobile platforms.

## 5 Conclusions

This study developed DE-HVT, a lightweight hybrid Vision Transformer that combines overlapping patch embedding, texture-sensitive central difference convolution, self-attention and convolutional feature modulation. Across three independent runs, DE-HVT achieved mean Top-1 accuracies of 93.51±0.44% on Plant Pathology and 99.82±0.06% on PlantVillage, with 4.10 million parameters and 0.85 GFLOPs. On the tested Snapdragon 865 platform, it required 46.86 MB of peak memory and recorded a maximum observed latency of 111.96 ms per image. These findings support the effectiveness of the complementary local–global design for fine-grained plant disease recognition and confirm its feasibility under the evaluated mobile-device conditions.

## Data Availability

Publicly available datasets were analyzed in this study. This data can be found here: https://www.kaggle.com/competitions/plant-pathology-2021-fgvc8 https://github.com/spMohanty/plantvillage-dataset.

## Supplementary material

Supplementary Table 1 Class-wise image distribution in the final evaluation sets.
