# Supplementary material for: Lightweight frequency-aware hybrid vision transformer for fine-grained plant disease recognition
Source: Front Plant Sci. 2026 Oct 13;17:1956762. doi: 10.3389/fpls.2026.1956762 (PMC13710256; doi:10.3389/fpls.2026.1956762)
Supplement: Supplementary file 1 [file Table1.docx]

| **Table S1** Class-wise image distribution in the final evaluation sets | | | | |
| --- | --- | --- | --- | --- |
| Dataset | Plant species | Disease/category | Total images | Share of dataset |
| Plant Pathology | Apple | healthy | 4,624 | 27.15% |
| Plant Pathology | Apple | scab | 5,712 | 33.54% |
| Plant Pathology | Apple | frogeye leaf spot | 3,346 | 19.65% |
| Plant Pathology | Apple | rust | 2,077 | 12.20% |
| Plant Pathology | Apple | powdery mildew | 1,271 | 7.46% |
| PlantVillage | Apple | Apple scab` | 630 | 1.16% |
| PlantVillage | Apple | Black rot | 621 | 1.14% |
| PlantVillage | Apple | Cedar apple rust | 275 | 0.51% |
| PlantVillage | Apple | Healthy | 1,645 | 3.03% |
| PlantVillage | Blueberry | Healthy | 1,502 | 2.77% |
| PlantVillage | Cherry | Powdery mildew | 1,052 | 1.94% |
| PlantVillage | Cherry | Healthy | 854 | 1.57% |
| PlantVillage | Corn | Cercospora leaf spot / gray leaf spot | 513 | 0.94% |
| PlantVillage | Corn | Common rust | 1,192 | 2.20% |
| PlantVillage | Corn | Northern leaf blight | 985 | 1.81% |
| PlantVillage | Corn | Healthy | 1,162 | 2.14% |
| PlantVillage | Grape | Black rot | 1,180 | 2.17% |
| PlantVillage | Grape | Esca (black measles) | 1,383 | 2.55% |
| PlantVillage | Grape | Leaf blight (Isariopsis leaf spot) | 1,076 | 1.98% |
| PlantVillage | Grape | Healthy | 423 | 0.78% |
| PlantVillage | Orange | Haunglongbing (citrus greening) | 5,507 | 10.14% |
| PlantVillage | Peach | Bacterial spot | 2,297 | 4.23% |
| PlantVillage | Peach | Healthy | 360 | 0.66% |
| PlantVillage | Pepper, bell | Bacterial spot | 997 | 1.84% |
| PlantVillage | Pepper, bell | Healthy | 1,478 | 2.72% |
| PlantVillage | Potato | Early blight | 1,000 | 1.84% |
| PlantVillage | Potato | Late blight | 1,000 | 1.84% |
| PlantVillage | Potato | Healthy | 152 | 0.28% |
| PlantVillage | Raspberry | Healthy | 371 | 0.68% |
| PlantVillage | Soybean | Healthy | 5,090 | 9.37% |
| PlantVillage | Squash | Powdery mildew | 1,835 | 3.38% |
| PlantVillage | Strawberry | Leaf scorch | 1,109 | 2.04% |
| PlantVillage | Strawberry | Healthy | 456 | 0.84% |
| PlantVillage | Tomato | Bacterial spot | 2,127 | 3.92% |
| PlantVillage | Tomato | Early blight | 1,000 | 1.84% |
| PlantVillage | Tomato | Late blight | 1,909 | 3.52% |
| PlantVillage | Tomato | Leaf mold | 952 | 1.75% |
| PlantVillage | Tomato | Septoria leaf spot | 1,771 | 3.26% |
| PlantVillage | Tomato | Spider mites (two-spotted spider mite) | 1,676 | 3.09% |
| PlantVillage | Tomato | Target spot | 1,404 | 2.59% |
| PlantVillage | Tomato | Tomato mosaic virus | 373 | 0.69% |
| PlantVillage | Tomato | Tomato yellow leaf curl virus | 5,357 | 9.86% |
| PlantVillage | Tomato | Healthy | 1,591 | 2.93% |
